# Inulin and Its Application in Drug Delivery

**DOI:** 10.3390/ph14090855

**Published:** 2021-08-26

**Authors:** Franklin Afinjuomo, Sadikalmahdi Abdella, Souha H. Youssef, Yunmei Song, Sanjay Garg

**Affiliations:** Pharmaceutical Innovation and Development Group, Clinical and Health Sciences, University of South Australia, Adelaide 5001, Australia; Franklin.Afinjuomo@unisa.edu.au (F.A.); sadikalmahdi.abdella@mymail.unisa.edu.au (S.A.); souha.youssef@mymail.unisa.edu.au (S.H.Y.); may.song@unisa.edu.au (Y.S.)

**Keywords:** inulin, drug delivery, nanoparticles, microparticles, hydrogels

## Abstract

Inulin’s unique and flexible structure, stabilization/protective effects, and organ targeting ability make it an excellent drug delivery carrier compared to other biodegradable polysaccharides. The three hydroxyl groups attached to each fructose unit serve as an anchor for chemical modification. This, in turn, helps in increasing bioavailability, improving cellular uptake, and achieving targeted, sustained, and controlled release of drugs and biomolecules. This review focuses on the various types of inulin drug delivery systems such as hydrogel, conjugates, nanoparticles, microparticles, micelles, liposomes, complexes, prodrugs, and solid dispersion. The preparation and applications of the different inulin drug delivery systems are further discussed. This work highlights the fact that modification of inulin allows the use of this polymer as multifunctional scaffolds for different drug delivery systems.

## 1. Introduction

**Figure 1:**
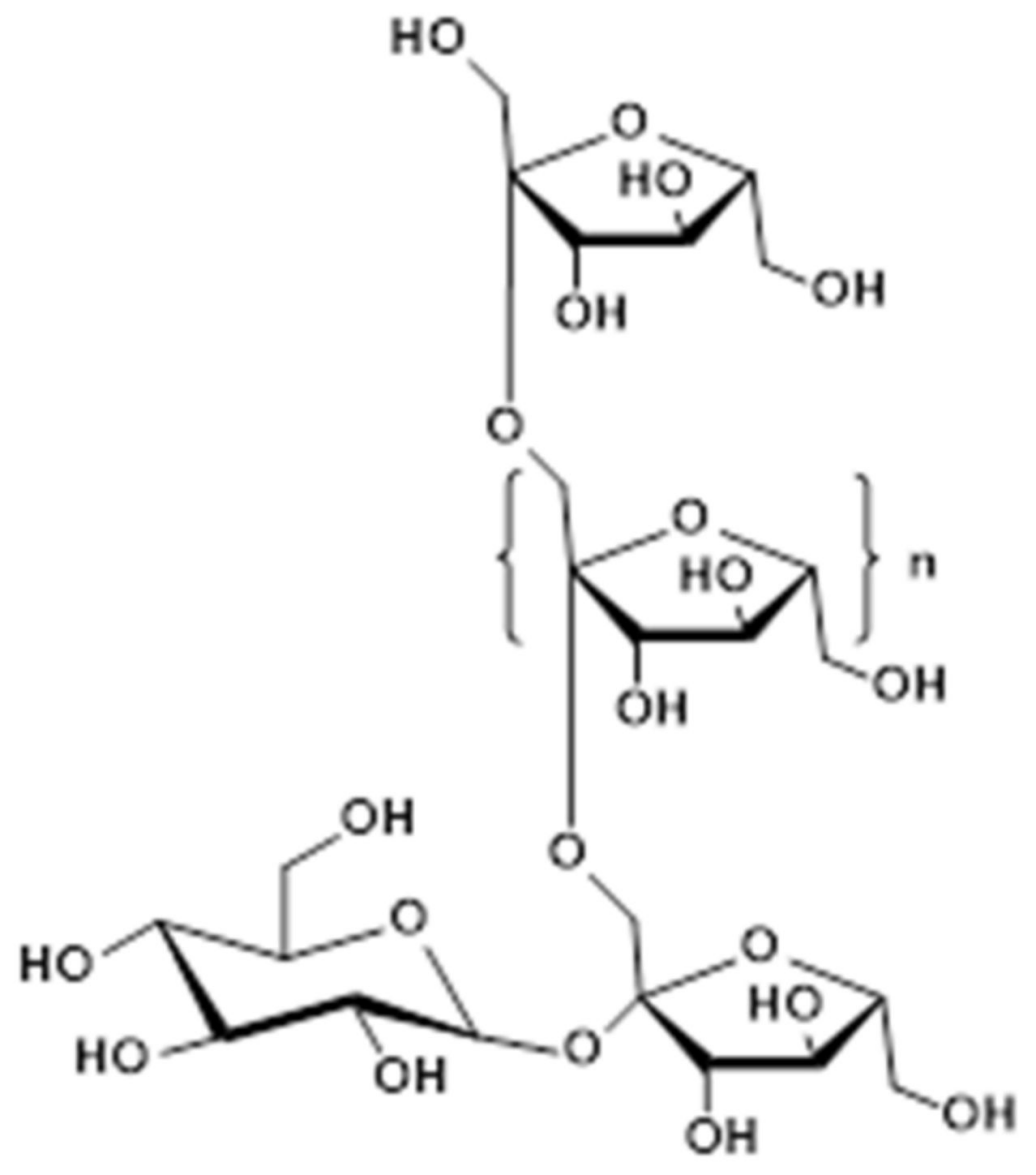
Inulin structure.

The use of natural polysaccharides for controlled drug delivery systems is drawing significant attention, as synthetics polymers used for this purpose are associated with drawbacks such as instability, expensive chemical modification [1] weak mechanical properties, poor receptor targeting properties [2], biodegradation, and excretion [3]. Natural polysaccharides possess properties that can address the multiple requirements for optimal drug delivery systems, including targeting and stealth properties [4]. In contrast to synthetic polymers, natural polysaccharides are non-toxic and biodegradable [5]. Inulin (INU) is a natural, highly abundant storage fructan polysaccharide that can be harvested from different plants such as chicory, dahlia, Jerusalem artichoke [6], vegetables, fruits, and grains, including garlic, leeks, bananas, onions, and wheat [7]. Genetically modified potatoes are also an important source of inulin [8] and enzymatic production [9]. The chemical structure of inulin is made up of linear chains of fructose units with a glucose unit linked to the reducing end [10]. The fructose units vary in length from 2–60 units. The variation in polydispersity and chain length can be traced to the plant species and time of harvesting [11,12]. The physicochemical properties of the polymer depend on the degree of polymerization (DP) and the presence of branching in the fructan [11]. Inulin has been used in drug delivery as a versatile polymer, and its versatility could be attributed partially to its flexible backbone. The flexibility of the backbone is, in turn, related to the absence of sugar rings in the polyethylene oxide-based backbone (Figure 1) [12]. In addition, the fructan is primarily made up of furanose groups. The molecular structure of inulin allows the polymers chain greater freedom to move as well as the ability to reassemble into a different range of structures [10,13]. This translates into structural flexibility and makes inulin an appealing and versatile material for use in different applications.

**Figure 2:**
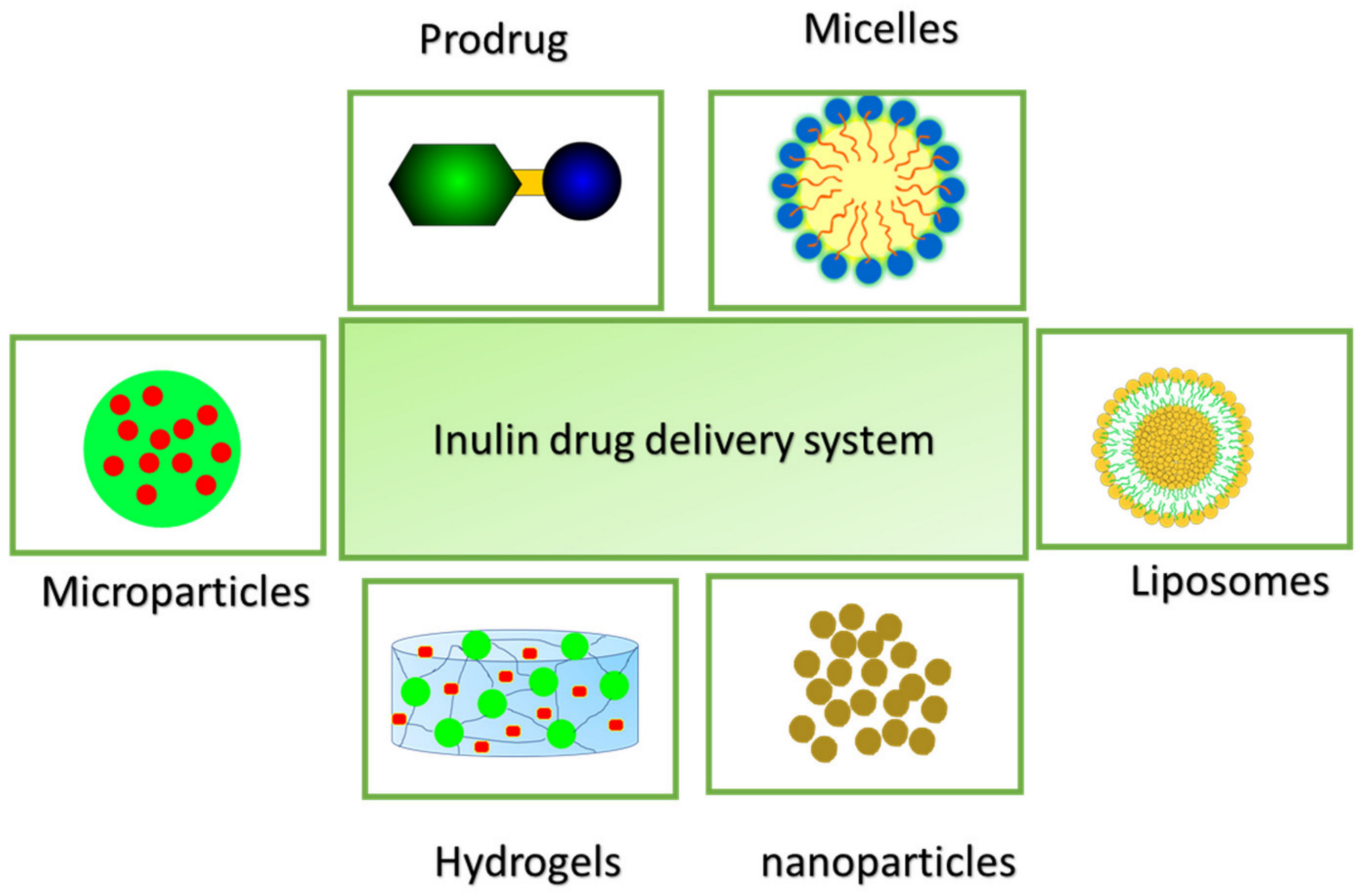
Inulin drug delivery carriers.

Another interesting feature of inulin’s structure is the β (2−1) glycosidic bonds that halt the polymer from being digested by endogenous enzymes such as other typical carbohydrates. Instead, inulin is relatively chemically stable in the gastrointestinal tract (GIT) until it reaches the colon, where bacteria in the gut digest it, making it a good source of carbon for health-promoting probiotic bacteria [14]. The change in solubility with chain length makes inulin suitable for different applications in both the pharmaceutical and food industries [13]. For instance, inulin with long chain lengths is insoluble at body temperature, making them useful for particulate delivery systems, while soluble (lower chain length) inulin is ideal for kidney and renal function testing [15]. INU is considered a non-toxic and highly biodegradable polymer. It is commonly used as a food ingredient and was given the Generally Recognized As Safe (GRAS) status by the United States Food and Drug Administration (FDA) in 2002. INU has also been used extensively in food industries as a sugar and fat replacement [15,16,17,18,19,20], nutritional excipient [21,22], texture modifier [15,23,24], and for organoleptic improvement [25]. The glycosidic bonds that humans cannot digest but that can be broken down by Bifidobacteria in the gut make inulin a suitable dietary fiber with prebiotic properties [10,26,27,28,29,30,31]. Aside from being degraded by inulinase that is found only in the colon, this polysaccharide passes straight through the stomach and the small intestine intact making it a good candidate for colon targeting. Inulin’s membrane stabilization ability is well established and has been used in pharmaceutical industries for several purposes, including to stabilize therapeutic proteins [32,33,34,35,36]. Modified inulin also improve the low dissolution of poorly soluble drugs [37,38,39], measurement of kidney function [40,41], modulate lipid production [42], glycemic control, as dietary fiber, prebiotic, treatment of constipation [43,44,45], vaccine adjuvant [46,47] and in cancer treatments [48,49,50] among others. Modified inulin has also been used for drug delivery [51] for targeting the colon [52,53,54] and pulmonary drug delivery [55]. Previous reviews by Barclay et al. [10] and Mensink et al. [13] explored the detailed physicochemical properties and pharmaceutical applications of inulin. Giri et al. also demonstrated the use of inulin derivatives for colon targeting [56]. The review by Jiwani et al. focused more on the delivery routes with emphasis on a few inulin deliveries forms [57]. The review by Akram et al. was not exhaustive and comprehensive because the review discussed few inulin delivery carriers [58]. Another good review from Wan and colleagues focused on inulin physiological functions and pharmaceutical uses only [59]. However, there is no detailed review focusing on the various types of drug delivery carriers such as liposomes, micelles, nanoparticles, microparticles, hydrogels, solid dispersion, and conjugates fabricated from the use of modified inulin. It is important to improve drug bioavailability, efficacy, reduce drug degradation as well as prevent serious adverse drug reactions. The improvement in the delivery of drugs helps minimize toxicities, improve patient compliance, and provide opportunities for pharmaceutical companies to market new products. Some of the delivery systems that have been successfully used to address delivery challenges include liposomes, micelles, hydrogels, nanoparticles, microparticles, microspheres, prodrugs, and solid dispersion. A review of these delivery systems will provide better insight into the use of inulin-based materials for researchers working in the area of pharmaceutical sciences. This gap was the reason and motivation behind the development of this inulin drug delivery review article. This review highlights the use of INU derivatives as multifunctional drug delivery scaffolds and vehicles. This review aims to fill that gap and draw attention to the huge potential of inulin as a multifunctional drug delivery scaffold (Figure 2). 

## 2. Inulin Drug Delivery Systems

The main focus of this review is to provide detailed insight into the various ways that modified inulin have been prepared and utilized for drug delivery applications. Inulin’s unique properties, such as molecular flexibility, ability to stabilize proteins, and self-assembled structures, ease of chemical modification, and immune modulation, encourage researchers and formulation scientists to exploit it as a drug delivery scaffold and biomaterials. Inulin’s ability to undergo chemical modification has been utilized to increase drug delivery to target cells such as cancers and genetic materials. The repeating fructose monomer has three hydroxyl groups (two secondary and one primary), which serve as a handle or platform to anchor other functional agents. Furthermore, Inulin stabilizes self-assembled proteins and lipid structures. For instance, it is noted that protein conformational changes and unfolding can result in loss of biological activity. To address this problem, there is a need to preserve the conformation by using protein stabilizers while formulating the products, which keeps the products safe during processing and for long-term storage [60]. One of the strategies commonly used for stabilizing proteins in vaccines and other pharmaceutical delivery systems involves adding polysaccharides. The abundant hydroxyl groups present in inulin allow the polysaccharide to replace interactions between water and proteins as they dry [61]. Additionally, the flexible molecular structure of the polysaccharide and the ability to form glassy polymeric arrangements with high glass transition temperatures facilitates adaption to the confirmation of the protein [62,63]. The reported mechanism of fructans providing membrane protection during drying includes inhibition of vesicle fusion and an increase in the lamellar spacing created by the polymer inserting deeply in between the layers [64]. Inulin demonstrated superior ability to stabilize proteins and lipid-based delivery systems when compared to many other polysaccharides due to the linear and flexible backbone [65,66].

For more than 60 years, low molecular weight, soluble inulin has been considered as the “gold standard” for assessing kidney function because it is predominantly eliminated by rapid renal excretion, making it a useful compound for measuring glomerular blood filtration rate [43,44]. The exceptional tropism of inulin towards the kidney makes this polysaccharide a potential carrier for kidney-targeted drug delivery. In addition to kidney targeting, many researchers have demonstrated the excellent colon targeting capacity of inulin. Colon targeting of drugs to the colon exploits the presence and variation of the bacteria population residing in various segments of the gastrointestinal tract. This is the basic concept of using polysaccharides degraded by colonic bacteria as a suitable carrier for colon targeting [67]. Inulin is particularly attractive for this purpose because it is not digested or absorbed in the upper GIT tract, whereas it is significantly hydrolyzed by inulinases produced by Bifidobacterium in the colon [65,66,68,69,70,71,72,73,74,75]. Inulin with a higher degree of polymerization (DP) is less soluble compared with those with lower DP. The difference in chain length of inulin can be used to tune solubility, which is then exploited for either particulate delivery systems (long-chain give insoluble inulin) or kidney and renal function testing (short-chain give soluble inulin). Inulin has been used in different drug delivery systems (Figure 2).

Different drug delivery systems:

- Hydrogel
- Micelles
- Liposomes
- Prodrugs/Conjugates
- Inulin complex/chelating
- Microparticles
- Nanoparticles
- Solid dispersion

### 2.1. Inulin Hydrogels

Hydrogel is a three-dimensional (3D) network obtained from hydrophilic polymers that can swell and hold a large amount of water or physiological fluid without dissolving [76,77,78,79]. Hydrogels maintain their structure due to the chemical or physical cross-linking of individual polymers. Among the delivery system and carriers available, interest in the use of hydrogel as biomaterials is growing rapidly due to their exceptional biocompatibility, biodegradation, good loading capacity, similarity to normal tissues in terms of physiological activity [80]. The fabrication of hydrogels can be carried out using either natural or synthetic polymers. Natural polysaccharides such as inulin, pectin, chitosan, hyaluronic acid, alginate, starch, gelatin, pullulan, dextran, guar gum are examples of polysaccharides that form hydrogels [81]. 

The functional groups present in the polymer structure, particularly the hydrophilic group such as hydroxyl, amine, and amide, confer on the hydrogel a good swelling capacity and ability to absorb water. In particular, the large number of hydroxyl groups in inulin provides a functional handle for chemical modification (Figure 1). Based on the method of fabrication, hydrogels can be either physically or chemically crosslinked hydrogels. The physical cross-linking of hydrogels involves weak forces such as ionic interactions, crystallization, and hydrophobic interactions between water-soluble polymer chains. The use of chemicals to crosslink the polymers results in chemical hydrogels [80]. Changes in external stimuli such as temperature, pH, or ion changes can provoke some form of physical crosslinking in hydrogel systems. However, some of these hydrogels are characterized by poor stability and mechanical properties. In contrast, hydrogels fabricated with the use of a crosslinker show remarkably better stability and mechanical properties. The disadvantage of these chemical crosslinkers is the cytotoxic issues, especially to the biological tissues. 

By tuning some parameters during the hydrogel preparation, the swelling behavior, crosslinking density, [82] mesh size, [77] permeability, mechanical strength, and drug release can be modified to tailor drug delivery to target tissues. By modifying inulin, stable hydrogels with desired physicochemical properties can be fabricated for use in drug delivery. Different approaches and strategies such as free radical polymerization of aqueous solutions of methacrylic anhydride or glycidyl methacrylates modified inulin [66,71,83], radical copolymerization, grafting polymerization [84], Michael addition crosslinking [85], UV radiation, chemical crosslinkers [86], enzymatic [87] have been reported for fabrication of inulin hydrogels. The use of aromatic azo agent bis (methacryloylamino)azobenzene (BMAAB) as a crosslinker for methacrylated inulin resulted in a rigid hydrophobic region in the inulin hydrogel [72]. Fabrication of smart inulin hydrogels that are responsive to environmental stimuli was using a different approach. For instance, addition reaction pH-sensitive inulin hydrogel was synthesized via chemical crosslinking with thiol-ene conjugate addition [54]. Modification of inulin with divinyl sulfone (DV), followed by succinic anhydride (SA), before crosslinking the derivative with tri-thiolated reagent resulted in the formation of hydrogel with the pH-sensitive property. The succinate groups confer pH sensitivity properties on the hydrogel [54]. Another pH-sensitive hydrogel was fabricated by initially modifying inulin with methacrylic anhydride and subsequently with succinic anhydride to form methacrylated/succinilated derivative (INU-MA-SA) followed by UV radiation to obtain the pH-sensitive hydrogel [88]. The use of radical copolymerization of modified inulin derivative MA-inulin with acrylic acid (AAc) in phosphate buffer solution with (pH 6.5) using both APS and TMEDA as an initiator reagent resulted in the formation of pH-responsive hydrogel [89]. Similarly, the condensation reaction between modified inulin derivative with the carboxyl group (INU–SA) and the hydrazide groups of water-soluble poly(amino acid) compound α,β-polyaspartylhydrazide (PAHy) with EDC and NHSS as coupling reagent results in pH-sensitive hydrogels [74]. Afinjuomo and his colleagues [86] fabricated pH-sensitive hydrogels by crosslinking inulin with an acidic anhydride-pyromellitic dianhydride (PMDA). They used the crosslinker (PMDA) via esterification reaction to synthesize hydrogels with pH sensitivity. The pH-sensitive hydrogels were useful and particularly important when considering target a specific area in the GI tract due to variation in pH along the gastrointestinal tract (GIT). While bioactive agents can be better protected due to less swelling at low acidic pH until the arrival of the hydrogel at the desired site (Colon). The extensive enzymatic degradation allows the drug encapsulated in the hydrogel to be released in the colon. Most of the reported pH-sensitive inulin hydrogels utilized both the change in the pH along the GIT as well as the presence of inulinase, which is predominantly found in the colon to achieved targeted delivery of drugs to the colon via the oral route. 

Afinjuomo et al. also reported the fabrication of novel injectable inulin hydrogels by using oxidized inulin and adipic acid dihydrazide as a crosslinker without the use of any catalyst or initiator [90]. The hydrogels are formed via Schiff-base reactions between oxidized inulin and adipic acid dihydrazide. In this case, the drug-loaded hydrogel shows enhanced in vitro release of 5FU in acidic pH compared to physiological pH. Inulin hydrogels are also formed via Michael addition crosslinking reaction between linear inulin and another crosslinking agent divinyl sulfone (DVS) in a sodium bis (2-ethylhexyl) sulfosuccinate (AOT) inverse microemulsion [91]. It is very clear from the above works that different chemical approaches have been devised for inulin hydrogel synthesis. However, the use of physical methods such as hydrogen bonding and supra-molecular chemistry needs further investigation. Cross-linking using enzymes and chemical synthesis in a water-friendly environment are also other possibilities that need attention. The fabrication of inulin hydrogels results in new biomaterials, which are subsequently exploited for the loading and encapsulation of different drug classes. Different classes of drugs such as NSAIDs, hormones, peptides, corticosteroids, anticancer, immunoglobulin G (IgG) [33], bovine serum albumin, and lysozyme. Inulin possesses great advantages and unique properties, especially for the delivery of drugs to the colon. This has been demonstrated in several drug molecules targeted to the colon. Loading of drugs into inulin hydrogel can be performed either by encapsulating the drug during crosslinking of the gel or permitting the drug to diffuse into the pores of the hydrogel after the crosslinking procedure [92]. Both methods have been reported for the loading of drugs into inulin hydrogel. Nonetheless, purification and removal of unreacted chemicals are very challenging with the encapsulation approach. The diffusion method involves adding the hydrogel into a drug solution and allowing it to swell to equilibrium. 

**Figure 3:**
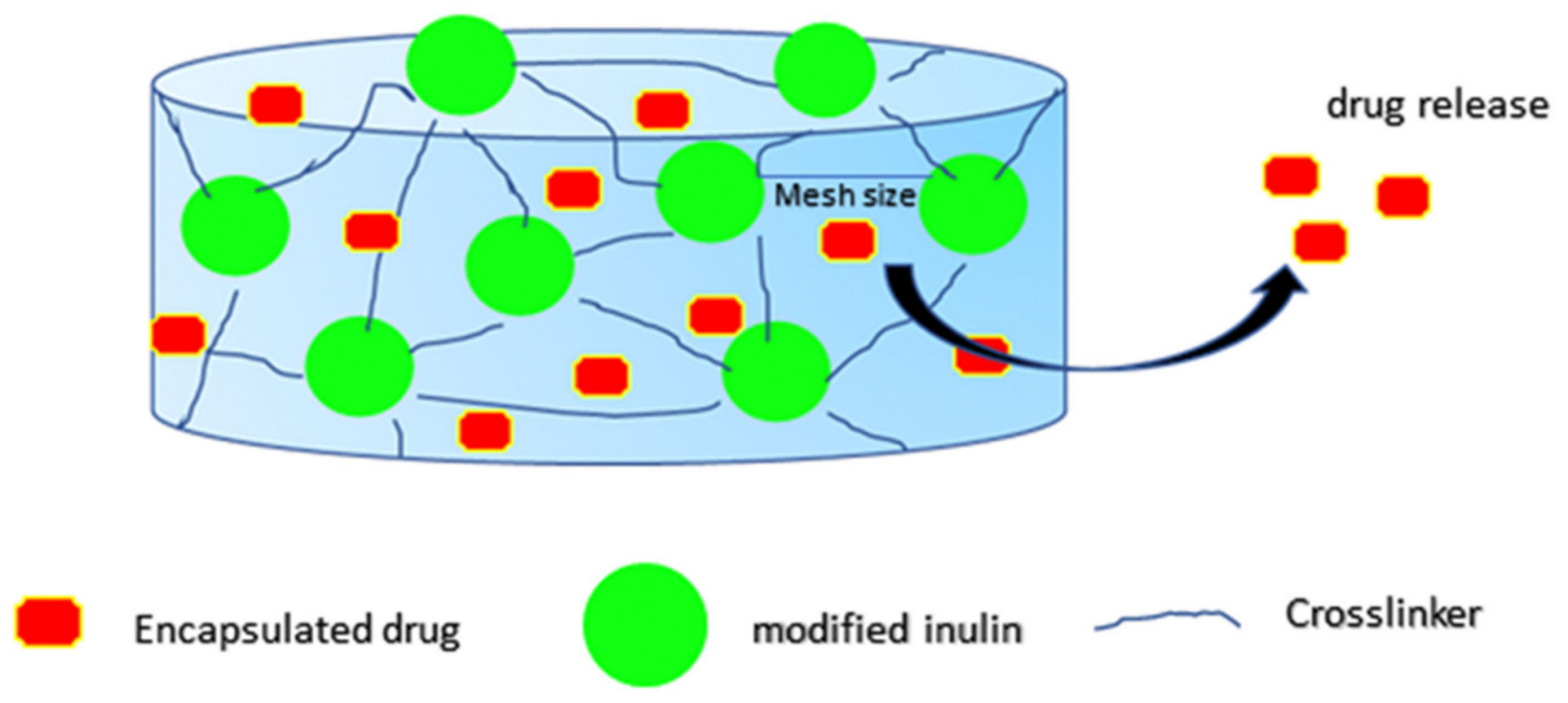
Hydrogel releasing of encapsulated drugs.

The in vitro release studies from pH-sensitive inulin hydrogels, under conditions mimicking the GIT, formulated for targeted colon delivery, illustrated that inulin could effectively protect the drugs from degradation in the upper part of the GIT, particularly in the stomach and intestine, and allow the release of the drugs in the colon. The release of active drugs from inulin hydrogel is assumed to be complex, and various release mechanisms have been proposed. Previous works suggested that three different release mechanisms, namely diffusion, swelling, and chemically controlled for the release of active drugs from the hydrogel [93]. The diffusion mechanism could be the principal way by which active drugs are released, considering the larger hydrogel mesh size compared to most small drug molecules (Figure 3). In this case, hydrogel mesh size in the swollen state allows diffusion of smaller molecules. Based on this mechanism, a macromolecules protein and peptide will likely have poor diffusion. In swelling controlled mechanism, the inulin hydrogel transit from glass to rubbery state, and the drug diffusion occur at a faster rate than swelling of the hydrogel. The slow swelling may be the rate-controlling process for drug release. The hydrogel experiences a swelling-driven phase transition from a glassy state to a rubbery state. Chemical control releases involve conditions where hydrolytic or enzymatic cleavage of the polymer chain or bond controls the release of drugs. Most of the inulin hydrogels reported in the literature seem to follow the swelling-controlled release. The driving force for drug release from hydrogel is the influx of solvent molecules into the hydrogel. In this case, the diffusion of drugs occurs at a faster rate than the swelling of the hydrogel. Two phases are formed during swelling, namely the inner glassy phase and the outer rubbery phase. Various parameters such as type of monomer [94], the molecular mass of peptide/protein [65,74], polymer concentration, the degree of substitution, loading concentration of the proteins [65], polymers swelling, and the electric charge can modify the release of drug/peptide from inulin hydrogel. For instance, the release of glutathione (GSH) and oxytocin (OT) from hydrogel synthesized from inulin INU–SA crosslinked with α,β-polyaspartylhydrazide (PAHy) was reported to be inversely proportional to the equilibrium swelling [74,95], which was also in agreement with reports from Maris et al. [72]. 

Factors that influence drug release from the hydrogels include loading procedure, degree of substitution, crosslinking density, pH sensitivity, and the type of bond. Van den Mooter et al. [65] prepared inulin hydrogel and loaded BSA protein into MA-IN hydrogel via two different loading methods (incorporation of proteins during crosslinking and after crosslinking). The authors reported that the hydrogel with BSA added during crosslinking exhibits a slow release pattern due to physical entrapment of the protein in the hydrogel compared with BSA loading via swelling. The swelling method results in protein release within one hour, which can be attributed to the inability of larger BSA molecules to diffuse to the inner smaller pore and the movement of the drug to the outer surface during drying [65]. A higher degree of substitution delays the diffusion of BSA from the hydrogel when compared to lower DS (15.4 versus 8.1). Crosslinking density is directly related to the degree of substitution. There will be less swelling with increased cross-linking density. The release studies of ibuprofen [75] and diflunisal [88] under conditions mimicking the GIT show pH-dependent release attributed to the carboxylic pendant group. 

Hydrogel from methacrylated inulin demonstrated a high swelling rate, which was significantly influenced by the degree of substitution, polymer concentration, and media ionic strength [70]. The mechanism of water transport in the swollen inulin gel is controlled by both inulin chain relaxation processes and diffusion (anomalous, i.e., diffusion and relaxation controlled) [70]. In this work, the media pH does not influence the swelling of non-ionic hydrogel. In contrast, pH-dependent inulin hydrogel formed between (INU–SA) and α,β-polyaspartylhydrazide (PAHy) exhibited lower swelling in acidic media. Due to the presence of ionic groups in the hydrogels, they showed greater swelling in water neutral pH compared to acidic pH. The carboxyl acid pendant in the hydrogel network confers and allows a pH-sensitive swelling. The reduced swelling of the gel in acidic media reduces the degradation of drugs in the stomach, making them ideal for drug delivery to the colon. The author reports protection of the encapsulated drug glutathione and oxytocin from the low acidic environment of the stomach [95].

The copolymeric compositions and crosslink density are other parameters that determine the degree of swelling of hydrogels. For example, in the formation of pH-responsive inulin hydrogels prepared from acrylic acid (AAc) and methacrylated inulin (MA-inulin) via radical copolymerization. The hydrogel demonstrated a sharp increase in swelling with an increase in pH from 2.2 to 7.4. At low pH, less swelling is observed due to the presence of the carboxylic acid pendant, but with further increase in pH, swelling increases due to the protonation of the carboxylic acid group [89]. Similarly, a pH-responsive hydrogel obtained from methacrylic anhydride modified inulin chains with succinic anhydride demonstrated a pH-sensitive swelling behavior due to the presence of the carboxylic acid pendant [88]. At low pH (1.0 and 4.0), the swelling ratio was low, but with an increase in pH (6.8 or 7.4), the swelling ratio was increased. The pH-responsive hydrogel synthesized from using PMDA crosslinker exhibited excellent and high swelling in water far higher than those previously reported for inulin pH-responsive [86]. This observation is justified by the electrostatic repulsion between the ionic group within the hydrogel network. For hydrogels with an ionic group such as carboxylic acid group/pendant, the ionization of the functional groups and dissociation of the hydrogen molecules within the polymer chain with change in pH play a key role in explaining why this hydrogel has reduced swelling in acidic media, which makes them ideal for drug delivery to the colon. 

Hydrogel biomaterials must be cleared from the human body after the delivery of drug cargo. One pre-requisite for the use of hydrogels in humans is biodegradability. The degradation of inulin hydrogels can be controlled and manipulated by different factors, including the type and cleavage of the bond in the polymer network as well as how the polymers are connected. Inulin is an attractive polymer for colon targeting because of the site-specific degradation of the glycosidic linkages of inulin by colonic microflora. Of note, it is important to investigate the degradation of modified inulin derivatives used for hydrogel formation by subjecting them to inulinase or cecal contents. 

The degradation of the modified inulin (INU-MA-SA) hydrogels after incubation with inulinase was investigated using High-Performance Liquid Chromatography (HPLC) and Thin-layer chromatography (TLC). The analysis of the result after 24 h showed the presence of a fructose spot on the TLC analysis, confirming the excellent biodegradation of inulin. About 53.3% of the hydrogel was degraded within a day, indicating that the crosslinking and functionalization of the inulin backbone did not prevent degradation of the inulin. In contrast, the radical crosslinking of N,N′-bis(methacryloylamino)azobenzene (B(MA)AB), and methacrylated inulin resulted in denser hydrophobic azo-inulin making accessibility to the inulinase enzyme very poor and difficult [96]. Bacteria strain present in the colon degraded dextran azo gel while inu-azo hydrogel showed less degradation. Adequate swelling is important for inulinase hydrolysis and degradation. Crosslinking agents can influence both the swelling and degradation behavior of inulin hydrogel. Excellent or sufficient swelling is necessary for the enzyme’s degradation of hydrogel in the colon environment. Detection of fructose monomers as a degradation by-product from hydrogels prepared by Pitarresi et al. [54,97] after incubation with colonic enzymes inulinase is another confirmation of the excellent biodegradability of inulin hydrogels. 

The degradation rate of inulin hydrogel decreases with an increase in the degree of substitution as more sites are required to be cleaved for degradation to occur. Increased crosslinking density of the gel makes them less degradable. It was noted that the amount of hydrogel degraded was directly proportional to the enzyme concentration. In general, by using modified inulin derivative, it is possible to tune or tailor the degradation kinetics of derived gels for colon targeting by using enzymes that are specifically localized in the colon.

Non-hydrogel Delivery (Beads, Vesicles, Pellets, tablets, and nano-hydrogel) systems.

The use of a non-hydrogel delivery system such as beads and vesicles [98] with inulin polymer as the starting material has been reported in the targeting of drugs to the colon. López-Molina et al. investigated the use of inulin vesicles obtained by solvent precipitation technique after modifying inulin with cinnamic acid chloride for the colon-specific delivery of methotrexate (MTX) [98]. The vesicles were biodegradable by inulinase. The release of the anticancer drug MTX was significantly increased after the breakdown of the inulin backbone by inulinase in cellular conditions [98]. Fabricated beads using natural polysaccharides inulin and alginate were successfully utilized to encapsulate three different probiotics strains [99]. The results from this work proved that the synbiotic formulations could prevent the premature release of the probiotics from the upper GIT and only permit release in the colon. The work demonstrated the potential of inulin to deliver live probiotics to the colon. Similarly, Bahadori and his colleagues [100] successfully synthesized nano-hydrogel loaded with 5-aminosalicylic acid (5-ASA) using negatively charged anionic Na-Alg nano-particles and coated with biodegradable quaternized inulin with 5-ASA encapsulated core-shell structured delivery system. The inulin component enhances the stability of the formulation in the upper GIT and only allows the release of 5ASA as inulin begins to degrade, resulting in the prolonged release of 5ASA. A new pH-sensitive phthalyl inulin (PI) tablet capable of protecting the probiotic bacteria from degradation from low gastric acidity (low pH) and only released the probiotics at the site of action was also developed [101]. The formulated tablets from phthalyl inulin (PI) show higher swelling in simulated intestinal fluid when compared to simulated gastric fluid. The difference in swelling was ascribed to the pH sensitivity of the inulin derivative. The formulated tablets with pH-responsive swelling are a promising approach for the oral delivery of probiotics. Hufnagel et al. [102] synthesized a novel pellet of inulin using hydrophobic and acetylated inulin derivatives. The inulin derivatives were subsequently loaded with 5-aminosalicylic acid. The pellets release about 80% of 5-aminosalicylic acid within 24 h under conditions mimicking the GIT intestinal environment. The developed formulations show promising results. The delivery platform can revolutionize the delivery of drugs during the treatment of Crohn and ulcerative colitis.

### 2.2. Inulin Micelles

**Figure 4:**
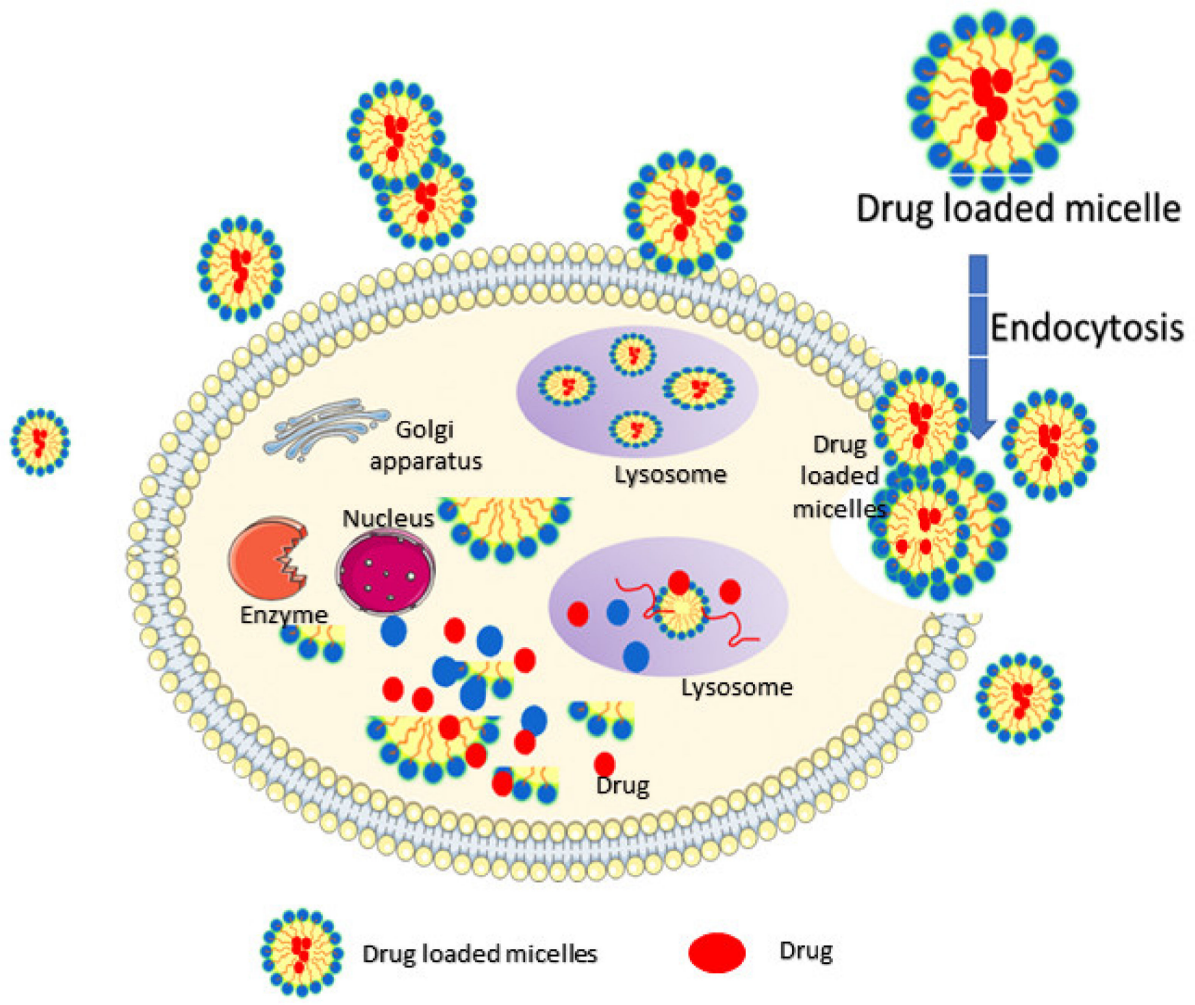
Polymeric micelle drug delivery concept.

**Figure 5:**
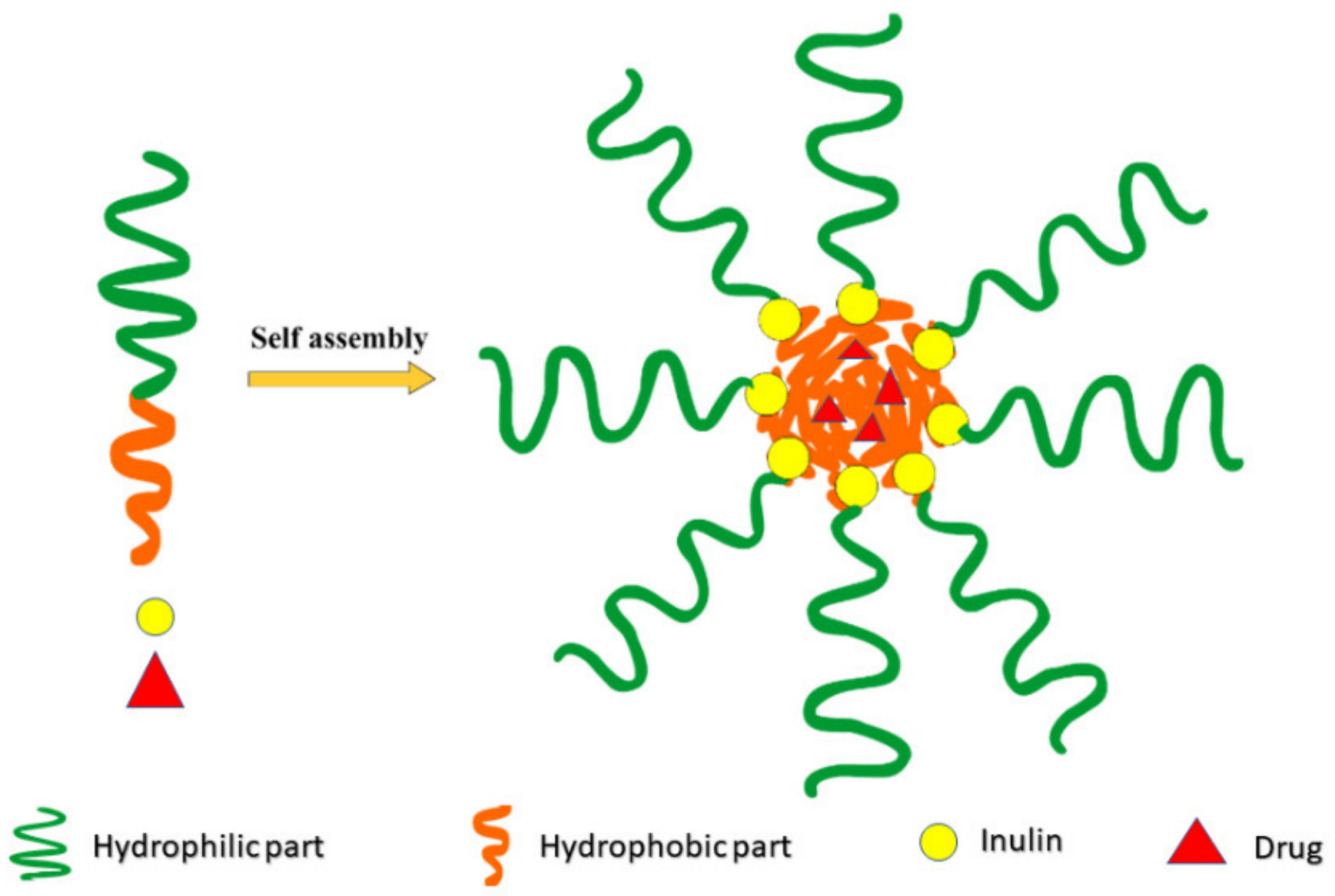
Inulin prodrug conjugate self-assembly into prodrug micelles.

Over the past two decades, the uses of polymeric micelles (PMs) as a versatile nanotechnology-based carrier system for poorly water-soluble drugs have gained tremendous attention [103,104,105]. The encapsulation of drugs with poor water solubility inside the hydrophobic core of PMs leads to significant improvement in solubility and bioavailability [106]. PMs are self-assembled core-shell nanostructures formed in an aqueous solution and consist of a hydrophobic core surrounded by a hydrophilic shell [106]. The accumulation of PMs into pathological tissues such as tumors and infracts with leaky vasculature is partly attributed to the small size ranging from 10–100 nm via enhanced permeability and retention (EPR) effect [107,108]. Interestingly, the small size of PM allows sterilization of micelle by filtration [109]. This system can be used for target drug delivery by modifying the outer surface using a specific ligand [110] (Figure 4 and Figure 5). The type of micelle formed can be influenced by the type of intermolecular forces driving the formation of the micelle. Based on these, three different categories of PMs have been reported, namely amphiphilic micelles, polyion complex micelles, and metal complexation micelles [111]. When amphiphilic molecules are exposed to an aqueous medium, there is spontaneous self-assembly into supramolecular core/shell structures consisting of a hydrophobic core surrounded by a hydrophilic shell.

**Figure 6:**
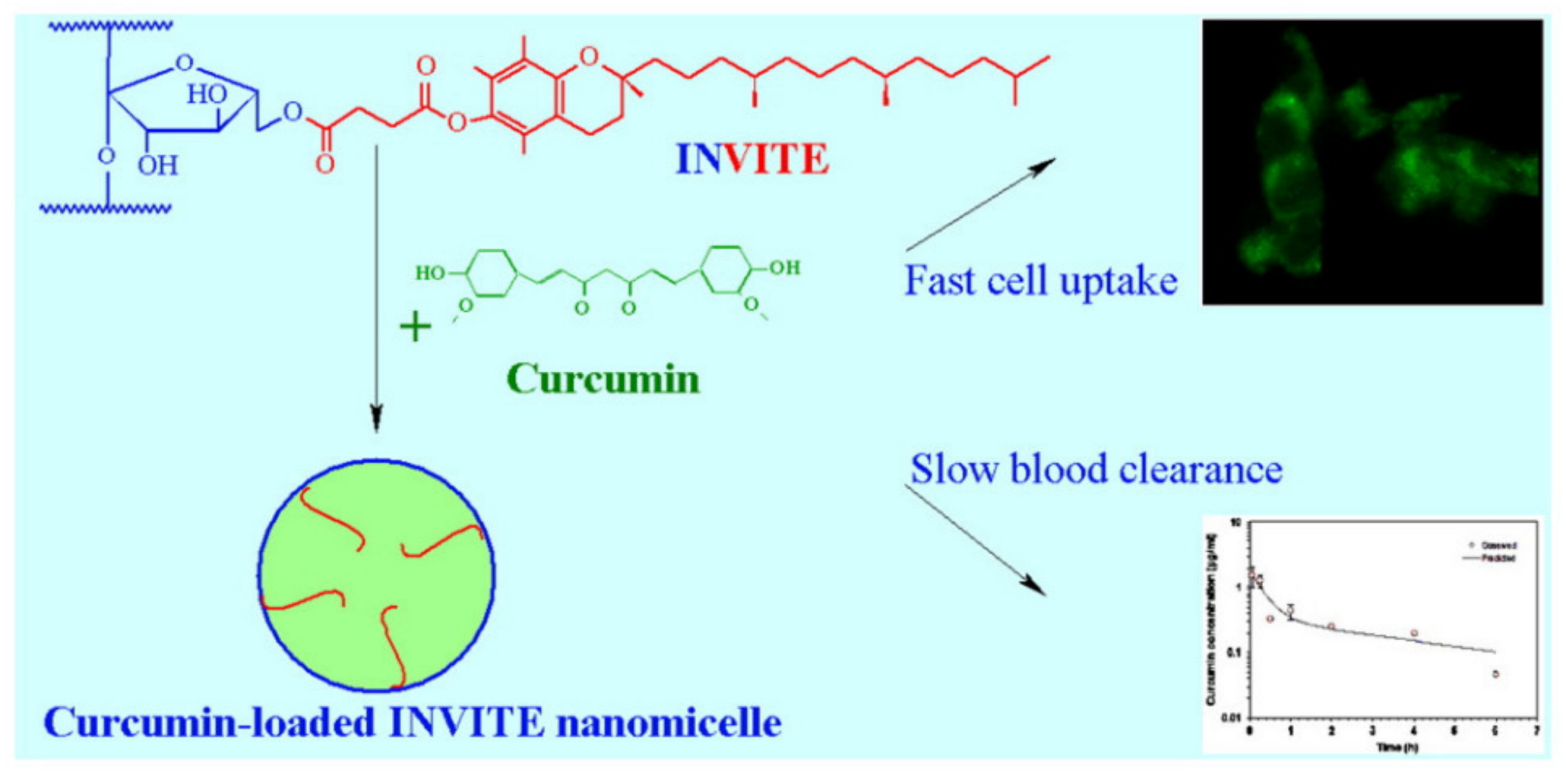
Inulin-D-α-Tocopherol succinate (INVITE) nanomicelles for intravenous administration of curcumin. Reproduced with permission [114]. Copyright 2015, American Chemical Society.

Different shapes of micelles can be synthesized, for instance, shapes such as spheres, rods, vesicles, tubules, and lamellae can be formed depending on parameters such as length of hydrophobic/hydrophilic blocks and solution conditions [112,113]. Most polymeric micelles use polymers such as inulin as the hydrophobic domain. The excellent biocompatibility, biodegradability, and non-toxic nature of inulin make it an attractive polymer for the formulation of polymeric micelles. Inulin derivatives such as inulin-α-tocopherol succinate conjugates (INVITE), an amphiphilic polymer, form nano micelles that helps to deliver hydrophobic drugs such as curcumin via intravenous route for anticancer use Figure 6 [114]. The delivery of combination drugs such as curcumin and celecoxib with poor water solubility was further investigated with the use of INVITE micelles for the control of angiogenesis in cancer.

The partial replacement of the hydroxyl anchor on inulin with VITE results in the formation of hydrophobized INVITE with the ability to self-assemble into stable nanomicelles at low concentration (from 2.4 × 10^−3^ to 22.3 × 10^−3^ mM) [106]. The authors reported that the inulin micelles improved the solubility of poorly water-soluble and highly hydrophobic drugs, including curcumin and celecoxib (CLX) [106]. The release, cellular uptake of curcumin into the tumor cell was enhanced by the use of INVITE-based nanomicelles. Micelles can also be used for the delivery of different drugs. For instance, Tripodo, G et al. [115] designed modified inulin functionalized with vitamin E (INVITE) micelles for the efficient delivery of rifampicin for the treatment of infections caused by mycobacterium tuberculosis. 

The modification of micelles surface with ligands can improve cellular uptake and increase the accumulation of the micelles in the target site. The potential use of polymeric INVITE micelles was further extended by decorating the surface of INVITE with biotin (BIO) to form INVITE-BIO micelles. This was then exploited for its long-circulating and targeting ability [116]. In addition, compared to INVITE micelles, the INVITE-BIO micelles demonstrated lower toxicity, long-circulating ability (48 h after IV administration), and improved drug accumulation in the tumor that over-express biotin through receptor-mediated endocytosis. In another study, inulin was modified with both hydrophilic chain poly-ethylene glycol (PEG) and hydrophobic ceramide to form a self-assembling amphiphilic inulin copolymer in water [117]. The micelle was then used to load and deliver doxorubicin, prolong blood circulation, and prevent recognition by the reticuloendothelial system due to the PEG component. The ceramide adds a hydrophobic effect and enhances antiproliferative signaling inside cells. The doxorubicin-loaded INU-ceramide-PEG2000 micelles demonstrated superior and preferential cytotoxic activity against cancer cells due to the enhanced uptake [117]. Similarly, Muley et al. designed a hydrophobically modified derivative of inulin (lauryl carbamate derivative of inulin). Inutec SP11 (INT) is another alternative to using a PEG block polymer in the delivery of paclitaxel via a micellar delivery system [118]. The resulting inulin amphiphile self-assembles in an aqueous environment with a low CMC of 27.8 μg/mL. The authors reported good hemocompatibility, low cytotoxicity, and enhanced antitumor ability in vitro and in vivo [118]. The formulation showed superior anticancer efficacy in mouse melanoma cells when compared with cremophor EL1 based Taxol formulation. In contrast to PEG, the INT micelles exhibited no production of antibodies or the accelerated blood clearance phenomenon, indicating that the INT micelles are safe carriers for IV administration of PTX [118] 

In another study, a novel amphiphilic inulin copolymer INU-ED-RA was synthesized by partial functionalization of the inulin hydroxyl group with ethylenediamine (EDA) followed by coupling of retinoic acid (RA) to the INU-ED via amide bond using carboiimide chemistry [119]. The hydrophobic copolymer self-assembles in an aqueous medium with particle size less than 500 nm to form stable micelles. It has been used for the encapsulation of corticosteroids such as dexamethasone, triamcinolone, and triamcinolone acetonide [119]. The micellar delivery systems when freeze-dried resulted in physical stability for about three months and are compatible with ocular cells. In addition, the micelles possess mucoadhesive properties and enhanced permeation of the corticosteroid into the corneal and epithelial cells. The micelle facilitates effective internalization and uptake into an ocular cell. The drug-loaded micelles efficiently deliver the model drugs in high amounts compared to free drug suspension, demonstrating the potential of this novel system as a carrier for corticosteroids in the treatment of a degenerative vascular disorder. 

On the other hand, smart lysosome-triggered delivery systems sensitive to pH environment that allow smart targeting of drug to tumor environment were synthesized using inulin. By using a four-step chemistry reaction, another pH-sensitive system, named INU-EDA-P, C-Doxo amphiphile was synthesized. Briefly, pentynoic acid was coupled to the modified inulin functionalized with amine via an amide bond, followed by attachment of the pH-sensitive citraconate pendants by reacting with citraconic anhydride. Lastly, dox was attached to the intermediate to form INU-EDA-P, C-Doxo using the ethyl-3-(3dimethylaminopropyl) carbodiimide (EDC)-mediated coupling reaction chemistry [120]. The final INU-EDA-P, C-Doxo prodrug conjugate self-assembles into prodrug micelles, which selectively release the linked DOX payload into the cancer cell cytoplasm. The inulin conjugates exhibited selective cytotoxicity against cancer cells. Interestingly, the conjugates self-assemble into organized nanosystems at low concentrations (0.33 mg mL^−1^) due to volume difference during trafficking between tissue and cells, which ultimately results in the preferential release of DOX into the cancer site [120]. The citraconylamide spacer confers pH sensitivity, which triggers and promotes selective release and accumulation of the DOX in the tumor cells compared to normal and healthy cells. The presence of doxorubicin and pentynoic pendants in the conjugates contribute to the π-π structure and hydrophilic/lipophilic balance necessary for self-aggregation [120]. It was recommended that this drug delivery concept needs further investigation because of the selective release of drug cargo once inside the lysosome via pH-dependent stimuli, making it a potential lysosome-triggered delivery system [121]. Chehardoli et al. [122] reported the use of inulin-grafted stearate polymeric micelle-based carriers (In-g-St), fabricated from the reaction of stearoyl chloride and inulin for the precise delivery of betamethasone to the damaged joints area. The authors highlighted that the smaller micelles from inulin-grafted stearate allowed the controlled release of a poorly water-soluble drug such as betamethasone. Micelles formulated from inulin derivative conjugated to the peptide via an ester linkage were successfully used for the encapsulation of Ornidazole for colon targeted delivery [123]. The amphiphilic inulin derivative was capable of self-assembly in an aqueous environment into nanosized biomaterials, which enable the loading and encapsulation of ornidazole. In this work, the author reported that the hydrophobic peptide arm supports and promote physical crosslinker, which resulted in self-assembly and formation of hydrophobic pocket and cores. However, despite the numerous advantages of polymeric micelles, there is a need for further research into stability and degradation.

### 2.3. Liposomes

Liposomes are drug delivery vesicles made of lipid that self-associate into bilayers with the ability to encapsulate the aqueous interior [124]. Liposomes offer several advantages, including circumventing the challenges of instability, cellular and tissue uptake obstacles, poor bioavailability, and poor in vivo biodistribution of drugs to the target site [125,126,127]. However, the setback to the use of the conventional type of liposomes as a drug delivery vehicle in clinical practice includes lack of specificity as well as recognition and degradation by the reticuloendothelial system (RES) due to their large particles size in the range of 500–5000 nm [128]. Novel approaches such as using smaller particles size (micro to nanosized scale) and surface modification with multifunctional carriers have been designed to improve the liposome drug delivery system [129,130,131]. Attaching targeting ligands to liposomes helped to specifically deliver the drug to target sites [132,133]. Inulin has been employed for sheath liposome strategy and as a component of the hydrophilic phase of the lamellar structure of liposomes. For instance, inulin has been used as a coating for liposomes and also for stabilization of labile therapeutic components in liposomes, polysomes, and lipoplexes [62,63,64]. They stabilized liposomes, providing opportunities for membrane interaction that results in increase acyl chain mobility and immobilization of the lipid head group regions due to their linear and flexible structure. Long-chain inulin conjugated to liposomes may give a dual advantage to the formulation (protect the drug from a harsh environment and increase the circulation time). Inulin derivative prepared by reacting oxidized inulin with ethylenediamine and diethylenetriaminepentaacetic acid and the liposomal encapsulation of this derivative with either 67Ga^3+^ or 111In^3+^ resulted in a stable complex in vivo [134]. The liposome-encapsulated inulin derivatives allowed loading and stability of heavy radioactive metal cations (111In^3+^ or 67Ga^3+^) for in vivo administration. Moreover, the liposomes were utilized for investigating variation in tissue distribution among different drug targeting or delivery protocols. The advantage of this liposomal system is that it provides a non-invasive method of studying the transport and delivery of drugs [134]. Inulin’s unique properties, such as macromolecular size, non-tissue distribution combined with the fact that it is not metabolized and only excreted in the urine, make the evaluation of intracellular drug delivery from the liposomal drug delivery system possible. 

### 2.4. Prodrugs

**Figure 7:**
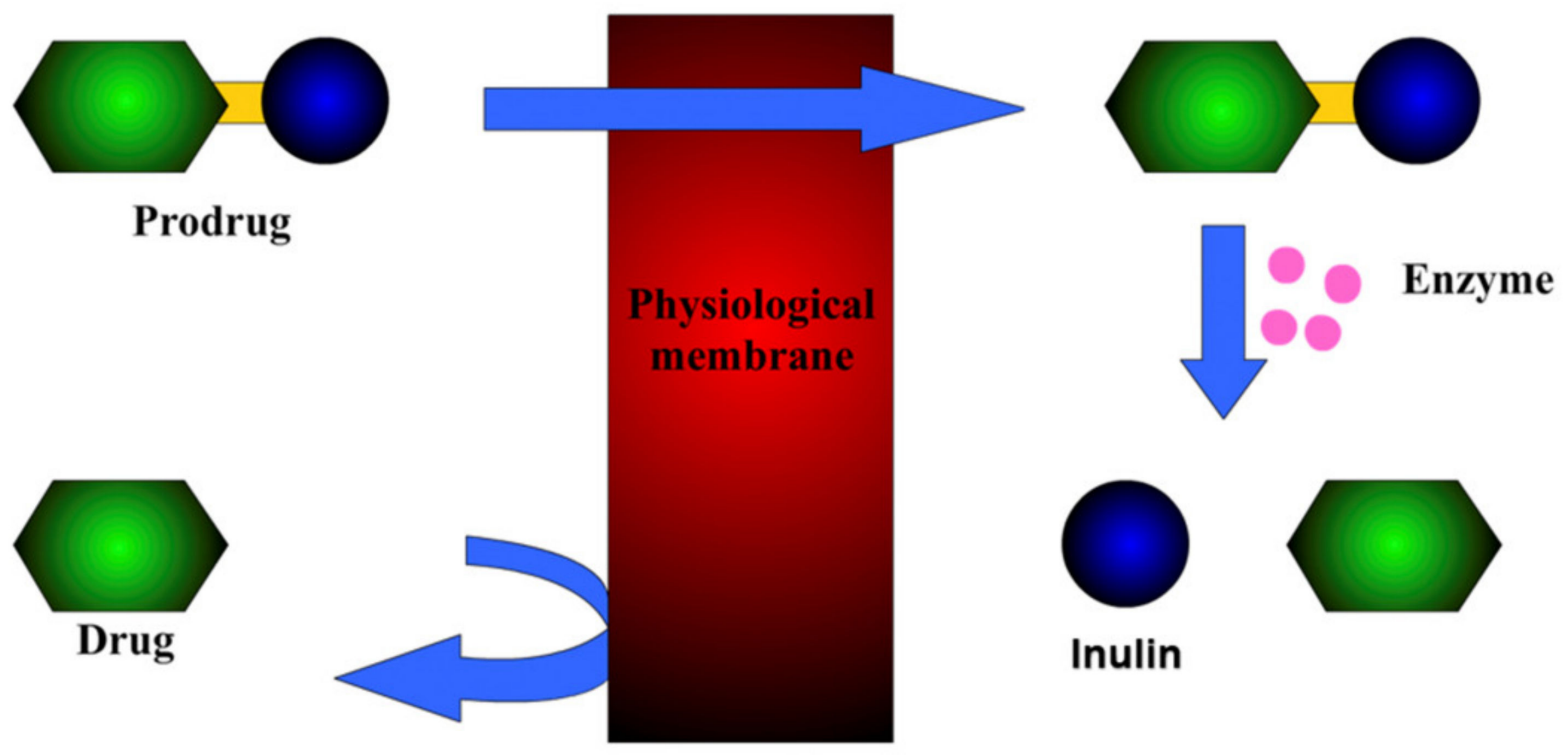
Diagram showing prodrug concept.

The design of polymeric prodrug is another strategy for improving the therapeutic application of drugs [135]. Conjugation of drugs to polymer is to improve poor bioavailability low solubility, [136,137], short plasma half-life, and chemical instability of drugs [138]. Furthermore, it masks the bitter taste [139,140], reduces systemic metabolism [141,142], and minimizes drug resistance [143]. Pharmacokinetic and pharmacodynamic properties of drugs such as inadequate blood-brain barrier permeability [144] and lack of selectivity at the site of action can be improved using a product [145,146]. The use of inulin conjugate is aimed at designing sustained-release formulations by increasing plasma half-life [147,148], targeting drugs to a specific organ, for instance, colon targeting [149], improve cellular drug uptake via pinocytosis, and reducing toxicity. Inulin therapeutic benefit, blood compatibility, non-toxicity, and biodegradability make it attractive and a suitable drug carrier using the prodrug approach (Figure 7). 

Modification of inulin by activating the hydroxyl group followed by nucleophilic attack and activation of carboxylic acid via EDC/NHS are two current strategies employed in the formation of the inulin prodrug. The two approaches result in esters and Schiff bases prodrug, respectively.

- Prodrug with an ester linkage
- Bioactive Schiff bases Prodrug

#### 2.4.1. Prodrug with an Ester Linkage

**Scheme 1:**
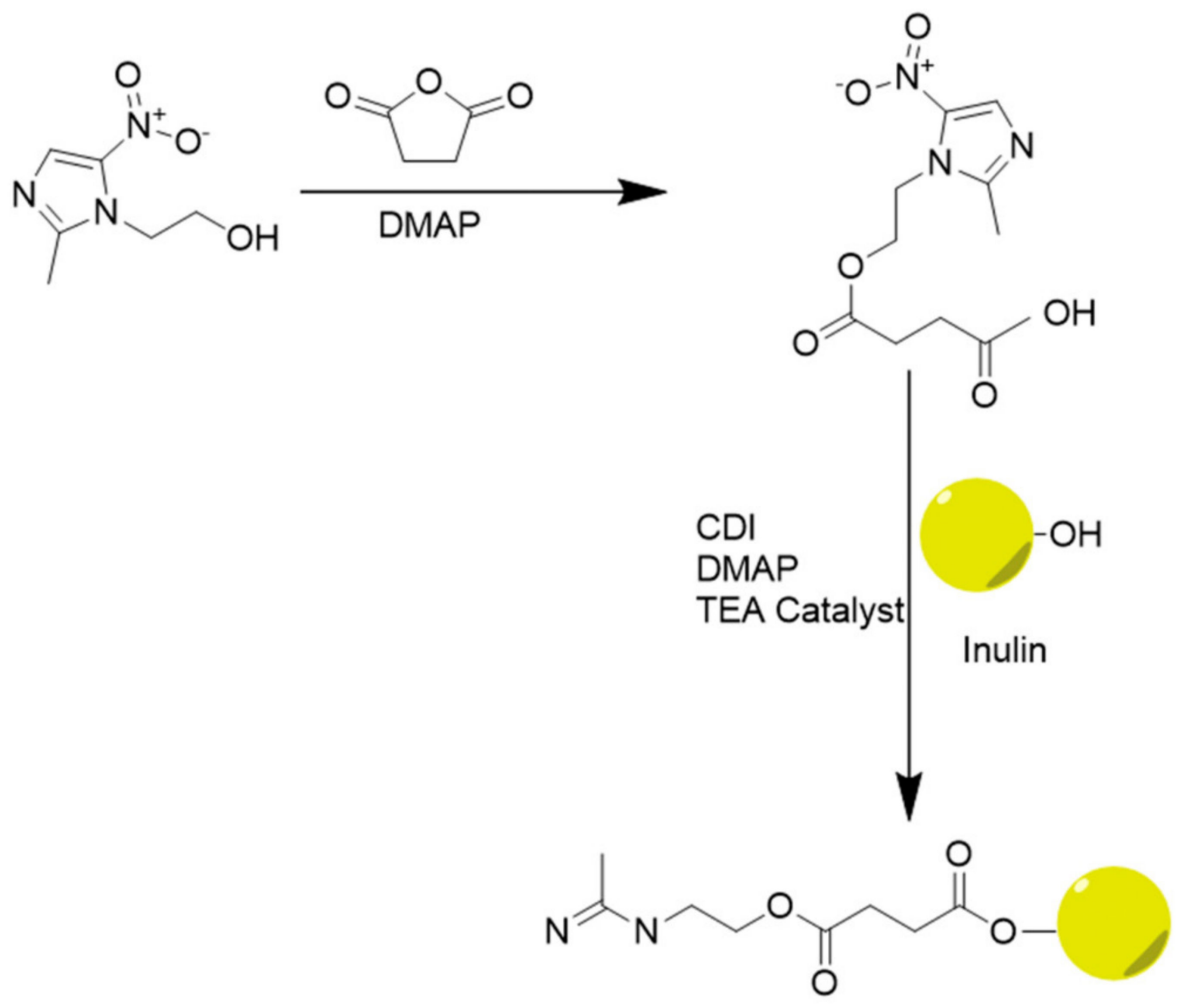
Esterification of metronidazole to form a prodrug.

Drug molecules with carboxyl and hydroxyl functional groups have poor bioavailability and limited diffusion across the lipid membrane due to ionization of the functional group under physiological conditions. The formation of inulin derivatives obtained by esterification reaction with other pro-moieties can give derivatives with the desired hydrophilicity, lipophilicity, and in vivo properties, as shown in Scheme 1. The rate of ester formation during the reaction can be increased by using either an acidic or basic catalyst such as 4-(dimethylamino) pyridine [150]. Metronidazole is the first-line drug for the treatment of anaerobic bacteria and protozoa infections, including amoebiasis. There is a need to target metronidazole to the colon for the treatment of conditions caused by the protozoa parasites. The parasite is also associated with complications such as hemorrhage and ulceration of the colon [151]. Vermeersun et al. reported the design of metronidazole inulin prodrug by succinylation of metronidazole to form metronidazole monosuccinate ester followed by coupling with inulin using 1,1′carbonyldiimidazole (CDI) chemistry. [152] (Scheme 1). As expected and illustrated in Scheme 1, the use of dimethylamino-pyridine (DMAP), triethylamine (TEA) with the coupling agent CDI improves the yield and esterification degree of the reaction. The inulin prodrug showed adequate stability in the acid environment of the upper intestine, but the prodrug was swiftly cleaved to release the drug cargo on incubation with the cecal contents suggesting that the enzymatic cleavage and degradation of the prodrug is vital for its bioactivation leading to amoebicidal action of metronidazole. The author suggests that the systems could serve as colon-targeted delivery systems for metronidazole in the treatment of amoebiasis [152]. 

Hartzell et al. synthesized an ester-linked conjugate using 5-ASA and inulin to form 5-fASA-inulin a potential prodrug for colon-specific delivery of both 5-ASA and inulin to the gut [149]. The release of 5-fASA from the prodrug was a significant increase in the presence of gut bacteria. However, the 5-fASA released was not hydrolyzable to active agent 5-ASA by the gut bacteria. The prodrug delays the inulin fermentation in the colon, ensuring the beneficial effect of polymer persists for longer in the colon. The non-conversion of 5-fASA to the active agent 5-ASA makes this system unsuitable for the intended drug delivery approach. Therefore, it is necessary to investigate an alternative approach for the conjugation of 5-ASA to inulin to produce biodegradable inulin derivatives.

#### 2.4.2. Bioactive Schiff Bases Prodrug

Amine-containing drugs often ionize under physiological pH, which facilitates faster diffusion, high first-pass effect, instability, and off-targeting of drugs into non-tumor sites [153,154], making the development of amine prodrug very challenging for formulation scientists. Developing a good amine prodrug was one way of overcoming this challenge. Schacht et al. [148], for instance, used the inulin prodrug of procainamide to improve its half-life and ensure a slow release of procainamide. In this work, the linkage of procainamide to inulin prevented the diffusion of the drugs to all parts of the body and ensured cellular uptake via pinocytosis. The conjugation involves oxidation of the hydroxyl group of inulin with sodium periodate, which resulted in the formation of dialdehyde followed by the coupling of procainamide via formation of the Schiff base and reduction with sodium cyanoborohydride. The procainamide moiety linked to the inulin backbone via hydrolytic imine conjugation bond was used to manipulate and tune the release of the drug. The in vitro release study showed that the drug was slowly liberated from the prodrug, and the rate was influenced by the ionic strength of the media [148]. The low yield of the prodrug was a limitation of this approach. The low yield can be attributed to the less reactive inulin hemiacetal intermediate from the oxidation process, which tends to reduce the effective attack of the intermediate by a nucleophilic agent. The problem of low yield was further addressed by using a different coupling method for the attachment of procainamide to inulin. In this case, inulin was activated by reacting with epichlorohydrin, and the derivative formed 3-chloro-2-hydropropyl was then reacted with procainamide in a neutral medium [147]. The author demonstrated that cellular uptake of the prodrug into yolk sac cells occurs via pinocytosis and the uptake process was dependent on the type of spacer and drug loading.

### 2.5. Inulin Chelating Agents/Complex/Polyplex

Magnetic resonance imaging (MRI) is one of the fastest and rapidly growing non-invasive diagnostic techniques that allow good and high-quality imaging of both healthy and diseased tissue/organ following the administration of a contrasting agent (CA) [155]. Currently, due to the high relaxation efficiency of Gd^3+^ -based contrasting agent, the vast majority of MRI scans are made after administering this CA [156,157]. However, Gd (III) toxicity in ionic form [157], long-term accumulation in the bone [158], and interference with protein and calcium-binding [159] limit its use for CA. In addition, this problem prevents the direct administration of this paramagnetic metal as contrasting agents (CA) for MRI. Hence, a complex is formed by attaching a ligand to Gd to overcome its toxicity. For this reason, one of the ways to address this limitation involves the formation of a low molecular weight Gd chelates/complex, formed by attaching ligands such as diethylenetriaminepenta-acetic acid (DTPA) and 1,4,7,10-tetra(carboxymethyl)-1,4,7,10-tetraazacyclodo-decane (DOTA). Unfortunately, the limited relaxivity (TR), rapid clearance, instability, short circulation time, toxicity, and precipitation of low molecular weight complex of Gd^3+^ limit their versatile application in MRI [157]. There was a significant compromise in the image resolution due to rapid elimination, making them unsuitable for angiography imaging.

As a result, there is a demand for novel contrast agents, which can address the above-mentioned limitations. According to the relaxation theory, an increase in the rotational correlation time can be achieved by linking low molecular-weight Gd^3+^ chelates to sugar polysaccharides, which automatically translate to higher relaxation rates [160,161]. To achieve this, inulin polysaccharide has been explored as a platform for the attachment of chelates and complexing agents. In addition, macromolecular MR contrasting agents have increased the blood retention time and increased relaxivity due to the increased steric hindrance that translates into slower molecular tumbling. A lower concentration of agents is required for acquiring a satisfactory image during MRI testing because the macromolecular structure permits higher loading of the chelates and metal ions [162]. Compared with protein, which can provoke an immunogenic reaction, sugar macromolecular carriers are less likely to cause this problem [163]. 

Different derivatives and approaches have been used to synthesized inulin macromolecular MRI contrast agents. Corsi et al. reported the design of a new inulin conjugate (aminopropyl)inulin (API)linked via diethyl squarate to the DO3A ligand (1,4,7,10-tetraazacyclododecan-1,4,7-triacetic acid) [164]. The high molecular weight (12,000 and 22,000 Da) of the two synthesized macromolecular conjugates with DS of 0.7 and 1.5, respectively, prevented diffusion into extravascular space and improved the blood circulation time. Inulin flexibility facilitates the formation of a derivative with a higher degree of substitution that ensures rigid Gd^3+^ conjugates, which ultimately contributes to increasing rotational-correlation time [165]. The efficacy studies, according to the report by the authors, show that the final complex Gd37[(API-(DO3ASQ) exhibits an increase in relaxivity and was also five times more potent than commercial contrasting agents at 20 MHz and 37 °C. Furthermore, the high relaxivity (20.3 mm^−1^ s^−1^) for this inulin complex gives credibility to its use as a promising contrast agent. 

To increase the rotational correlation time, Lebduskova et al. designed a novel inulin conjugate and a Gd (III) chelate by covalently attaching carboxylic-phosphorus acid derivative of diethylenetriamine with inulin [166]. The novel agent formed in this work allowed the replacement of the central carboxylic pendant arm of 1,1,4,7,7-diethylenetriaminepentaacetic acid (H5DTPA) with a phosphinic acid functional group to form an intermediate, which was then subsequently linked to O-aminopropylinulin. The efficacy of the novel macromolecular contrast agent was evaluated at 20 MHz and 37 °C and the resulting NMRD profile showed that the inulin derivative was four times more potent than the commercially available contrast agents. This is mainly due to an increased rotational correlation time (866 ps at 298 K). The kinetic stability of the novel macromolecules was similar to the clinically approved MRI contrasting agent [Gd (DTPA)(H_2_O)]2^−^. Moreover, the inulin macromolecular structure allows the attachment of about 24 Gd^3^+ ions per molecule, meaning lower concentration can be used for MRI enhancement. Toxicity will also be reduced as a result of using low concentration, which would help to increase MRI enhancement. The author reported that the final conjugate molecular weight of about 23,110 partly contributes to the favorable residence lifetime. 

However, the poor kinetic stability of the previous contrast agents based on inulin macromolecules prompted Granato and his co-workers to design a stable Gd^3^+ complex by conjugating modified inulin derivative O-(aminopropyl)inulin with DOTA-NCSA[1,4,7,10-tetraazacyclododecane-1,4,7,10-tetraacetic acid mono(p-isothiocyanatoanilide)] [167]. This Gd–inulin complex displayed superior kinetic stability compared to the conjugates prepared previously using a carboxylic-phosphorus acid derivative of diethylenetriamine [166] and API-DO3ASQ conjugates [164]. The relaxivity at 20MHz and 37 °C for these contrasting agents was higher than the model compound (21.7 vs 5 s^−1^ mM^−1^), and this was attributed to reduced molecular tumbling originating from attachment to the inulin carrier.

The functionalization of the conjugates with targeting moiety improves the specificity of the CA by promoting vascular confinement to compromised tissue only. The rotational correlation times at 37 °C for the GdLx–API conjugate is about 17 times higher compared to GdLx. The authors reported that both GdLx and GdLx–API conjugate did not interact with human serum albumin. The high molecular weight of the CA macromolecule contributes to an increasing plasma circulation time, making it suitable for cardiovascular angiographic imaging. Inulin serves as an excellent carrier due to its flexibility [162,164,166,167] that allows the formation of inulin derivatives with high DS. The variable molecular weight of inulin 1500 and 5000 Da (depending on the degree of polymerization (10–30) support the formation of the derivative with a high molecular weight macromolecule suitable for use as a contrasting agent. Other properties of inulin, such as low viscosity, inertness, and not being metabolized in the blood, make it ideal for CA application [167]. The use of different tethers can influence the rotational correlation time of the CA. For instance, DO3A ligand, H5DTPA, and DOTA-NCSA when attached to inulin have rotational correlation time of 735, 866, and 1460 ps, respectively, at 37 °C, and the difference can be attributed to the use of a different tether to the same inulin backbone, which ultimately affects the mobility and rigidity of the final product. The excellent biodegradability of inulin coupled with its macromolecular size limits plasma clearance and promotes vascular confinement to cancer or diseased tissue. This makes inulin complexes ideal and more efficient contrast-enhancing agents than low molecular weight complexes. Nonetheless, the slow clearance of inulin complexes from the body may result in Gd^3+^ ions accumulation and toxicities [157]. From the studies above, it is clear that Gd- inulin complex is efficient, but there is a need to prove the safety of the Gd- inulin complex to realize its full potential in CA application. Short interfering RNAs (siRNAs) have been used in silencing the expression of genes that are involved in the progression of diseases such as cancer, cardiovascular, neurodegenerative, metabolic diseases, and viral illnesses [168]. The effective cellular uptake is obstructed due to their inherent negative charge and poor diffusion across the membrane. In addition, within the endosomes, there is the sequestration of the gene, which reduced the number of active drugs. Even though cationic polymers such as PEI exhibits high siRNA condensation and transfection ability, their full potential use is limited due to their non-biodegradability and cytotoxicity. All of these points further establish that there is a need for an improved delivery system.

Different strategies have been employed to overcome degradation, cellular uptake, and off-target problem. The use of cationic polymer is a very common and effective way for gene delivery because the polyplexes formed between the cationic polymer and nucleic acids result in reduced degradation and endosomal escape while improving cellular uptake. The use of functionalized and modified inulin represents one approach to overcome the toxicity obstacle without the need for extensive and multiple-step chemical modification. Different functional groups such as ethylendiammine (EDA), diethylediamine (DETA), spermine (SPM) have been conjugated to the inulin backbone [169]. Polycation synthesized by conjugation of inulin with diethylenediamine possesses positive charges at physiological pH, which allow its use as a carrier for siRNA [170]. The Inu-DETA copolymer can bind and release the siRNA in JHH6 cells with less cytotoxicity. Further modification of the INU-DETA imparted improved properties in the biomaterial. The introduction of imidazole (IMI) to the already synthesized INU-DETA copolymer results in the formation of INU-IMI-DETA, which exhibits pH-dependent response as well as strong cationic characteristics [171]. This new derivative demonstrated better cellular uptake due to self-aggregation into Inulin Complex Nanoaggregates (ICONs) triggered activated by physiological salt concentration and pH elevation [171]. ICON shows a superior ability to condense a higher amount of siRNA molecules because the protonation of the imidazole group will prevent escape from the endosomal/lysosomal compartment. The functionalization of the INU-IMI-DETA for active targeting are potential strategies that need further exploitation for future gene therapy delivery. The search for new biocompatible and safe inulin derivatives for gene delivery led to the use of the atom transfer radical polymerization (ATRP) approach to graft poly (DMAEMA) onto the inulin backbone [172].

The inulin cationic copolymer formed a strong complex with RNA/DNA via electrostatic interaction. The author reported that a low hemolytic rate and high transfection efficiency highlight that the modified inulin copolymer (DMAEMA)(PDIN) might be an excellent biocompatible candidate for gene delivery systems. The transfection efficiency of this copolymer was three times higher than that of the popular lipofectamine 2000 transfection reagent. The possibility of using modified inulin derivatives for the delivery of iron as well as iron supplements in the diet was demonstrated by Pitarresi and his colleague’s findings [173]. The complex formed between iron, carboxylated, and thiolated inulin derivative (INU-SA and INU-SA-Cys) was exploited for the oral delivery of iron. The authors reported that the release profile of iron from the copolymer delivery system in a condition mimicking the intestinal tract was excellent, and the derivative also demonstrated good mucoadhesion properties [173]. In addition to the release, the modified inulin was broken down by inulinase despite modification.

### 2.6. Microparticles

Inulin microparticles have been used to deliver drugs, proteins, vaccines antigen, and other agents by adsorbing, attaching, or entrapping. Depending on the desired physiochemical properties, inulin microparticles can be prepared by either chemical or physical methods. The desired particle size and chemical properties of the drug/active agents determine the technique to be used. Inulin biodegradability is important when used as microparticles because the drug cargo entrapped in the matrix is released when the particle degrades. Furthermore, another good reason for using inulin can be attributed to its FDA-approved status, and the hydroxyl group in the structure provide a chemical handle and platform for modification. The degradation rate and drug release can be tuned by varying some parameters during the synthesis of inulin microparticles. In addition, the ability of inulin particles to be functionalized with targeting ligand makes it an excellent and ideal carrier. Modification of inulin results in derivatives with improved properties without changing the fundamental skeleton of the inulin backbone. Inulin microparticles have been reported as a carrier and adjuvant for vaccines. For instance, apart from stabilizing vaccines, delta inulin microparticles were reported to exhibit potent immunomodulatory properties making them suitable adjuvant [174]. Incorporating antigen into inulin microparticles resulted in targeted delivery of the antigen to the APC as well as adjuvant effects (dual function) [175,176]. By using special incubation conditions, seven different isoforms of inulin were fabricated by Silva and co-workers [46], but only the γ-inulin and higher isoform, insoluble semicrystalline particulate forms at 37 °C, possessed highly potent immunomodulatory properties [46,174]. Inulin semicrystalline particles, especially the delta inulin, showed promising results both as adjuvant and delivery systems for the vaccine [177]. The image characterization of these semi-crystalline forms of inulin using SEM and AFM morphology studies showed that they are regular discoid structures made up of piles of lamellar sheets with a diameter of about 1–2 μm, which facilitates their uptake by monocytes [177]. This unique size of Delta inulin (γ-inulin) microparticles allows efficient internalization by antigen-presenting cells (APCs). In addition, the excellent tropisms of these particles towards monocytes and dendritic cells are partly attributed to the surface molecular organization of the particles. In contrast, the soluble inulin does not possess immunomodulatory properties.

Another distinctive advantage of γ-inulin (delta inulin) microparticles is the ability to stimulate both the humoral and cellular immune responses without activating NFKb or inflammatory response [178]. These microparticles share a different mechanism when compared to other polysaccharide adjutants. Delta inulin activates the complement via the ACP, unlike other polysaccharides such as dextran, mannan, chitin, and glutan, which activate NFKb and inflammatory response [179,180,181]. Delta inulin remains a strong candidate for human vaccine adjuvant development because it is highly efficacious, potent, safe, and well-tolerated both in human, animal young foals, and pregnant mares [182,183].

In recent times, the use of soluble inulin as an adjuvant was reported. However, the soluble inulin particles in this work did not demonstrate potent immunomodulatory properties similar to the delta inulin polymorph [184]. Water-soluble inulin with particle size of 1.5 ± 0.12 μm was exploited as a drug delivery carrier for ovalbumin and as a vaccine adjuvant [184]. The ovalbumin-loaded inulin microparticles (SIMs) were synthesized by the emulsion–precipitation technique. Characterization of the microparticles by SEM shows that the particles are spherical in shape and the average diameter is about 1.5 μm promoted the uptake of the antigen into the APC’s. Mice immunized with SIMs show significantly higher antibody titers and enhance cellular uptake of ovalbumin antigen by APC compared to alum plus ova or ovalbumin alone. The burst release of antigen was minimal (20% in less than 30 min) compared to about 50% release in 5 min with inulin microparticles encapsulated with BABCH. The microparticles allow the release of ovalbumin in a controlled manner, with about 70% of the ovalbumin released over 16 h. Unfortunately, the SIM shows poor Th1 type immune response, and this may require the use of agents such as CpG, MPLA, which can help increase the immune response to address this limitation. Another strategy used for the fabrication of microparticles was the emulsion/solvent evaporation method. Gallovic et al. evaluated the potential of soluble inulin microparticles with a diameter greater than 250 nm produced from acetylated inulin by emulsion/solvent evaporation method as carrier and vaccine adjuvant for ovalbumin (OVA) antigen [185].

This work exploited the change in pH along the phagocytic pathway to trigger the release of loaded antigen and immunostimulatory hydrolytic by-products from acid-sensitive acetylated inulin microparticles. The result shows that the inulin microparticles were able to induce a variable and significant amount of TNF-α production compared to unmodified inulin due to the cellular uptake of the Ace-IN microparticles by APC. Following immunization in mice, there was higher antibody production in mice administered with ovalbumin encapsulated inulin microparticles compared to pure soluble ovalbumin. This work demonstrated that inulin microparticles can efficiently deliver ovalbumin to APC as well as act as an adjuvant. The degradation of the acetylated inulin can be tuned and manipulated to the desired parameter. For example, varying the reaction time during the synthesis of acetylated inulin (from 20 min, 1 day to 3 days) results in the formation of 0.4, 20.4, and 26.7% of cyclic acetal coverages, which provide a chemistry platform to tune the degradation of the acetylated inulin. The ability to control the degradation of acetylated inulin allows the control of the immunostimulatory and TNF-α production [185]. The large size of this microparticle still allows passive targeting to the APC.

The acid-labile modified inulin makes it pH-sensitive, thereby preventing burst release until it reaches acidic phagolysosomes of APCs and also, the production of neutral degradative by-product gives these derivatives some advantage as a suitable carrier for antigen. It is worthwhile to point out that acetylated inulin particles were recently exploited as both adjuvant and antigen delivery vehicles against infectious disease and cancer [186]. The modified acetylated inulin inability to activate cells with TLR-4 antagonists and mice lacking TLR signaling support the assumption that they are toll-like receptor-4 (TLR-4) agonists. The encapsulation of acetylated inulin particles with antigen confers the ability to imitate pathogens, further translating into better antigen delivery (about 47 times more potent) compared to soluble antigen alone.

**Figure 8:**
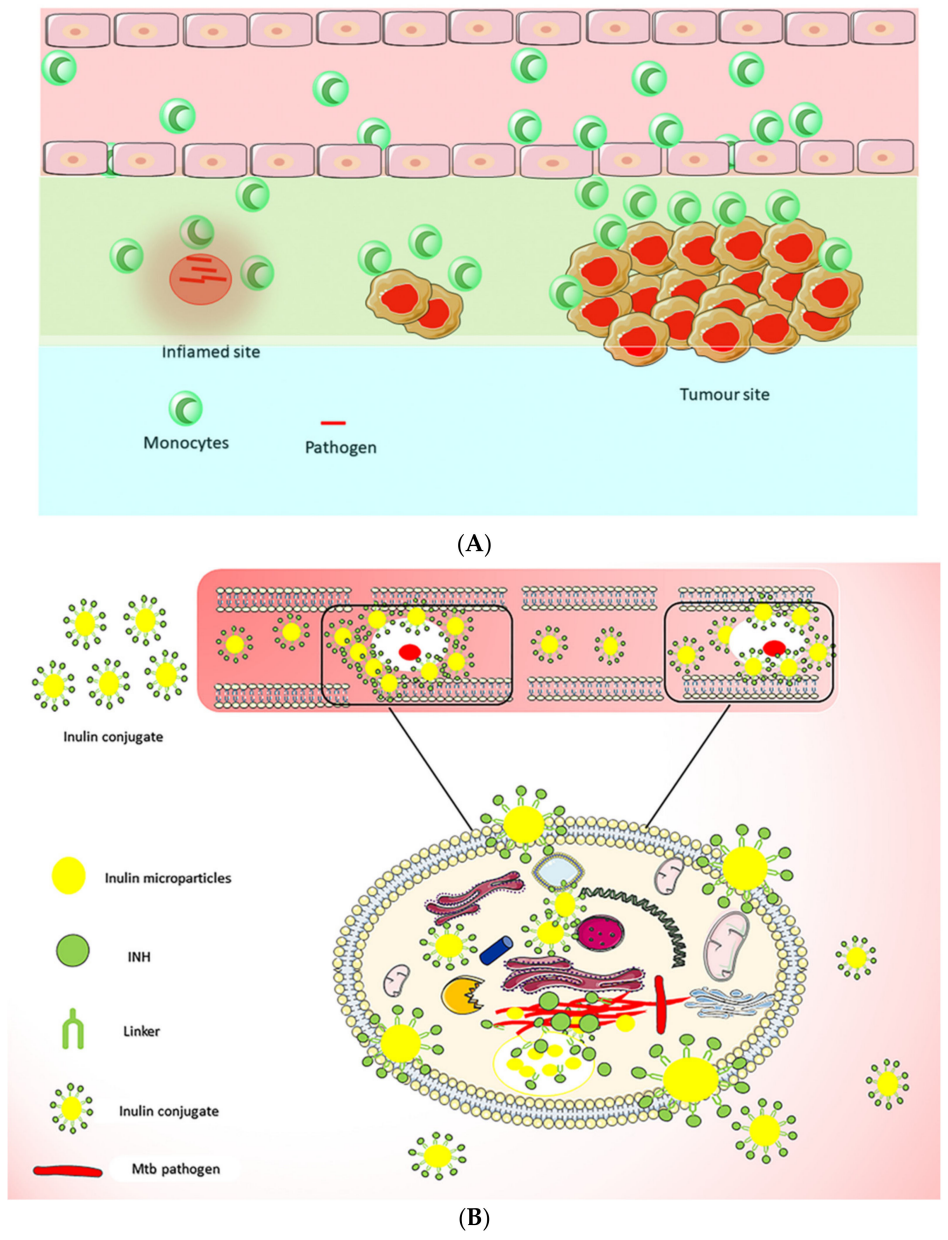
(**A**) schematic diagram showing monocytes targeted delivery to inflamed and tumor site. (**B**) Isoniazid intracellular targeted delivery to infected monocytes with inulin microparticles. Reprinted from [187].

It is also possible to use engineered inulin microparticles to target monocytes and macrophages, which can be useful in the treatment of inflammatory disorders, tuberculosis, AIDS, and other infectious diseases. Afinjuomo et al. [187,188] used Delta inulin particles that were engineered via conjugation chemistry to efficiently deliver two different antitubercular drugs, namely pyrazinoic acid and isoniazid to host macrophages. They showed that the synthesized inulin conjugates could be used for intracellular delivery to the tubercle bacilli reservoir (Figure 8). The modified inulin microparticle shows a diameter of about 1–2 μm from the SEM results, which help contributes to the excellent uptake and tropism towards monocytes and dendritic cells.

Overall, the result demonstrates that the inulin delivery system can target the monocytes/macrophages harboring the intracellular Mtb pathogens (Figure 8B) [187]. Apart from infectious disease, the use of delta inulin was further exploited to deliver and promote uptake of anticancer drug doxorubicin to the monocytes [189]. This delivery system was also utilized to target doxorubicin to the lymphoid organs. The authors demonstrated that pH-dependent release for the conjugate as well as cellular uptake.

An alternative approach was reported for synthesizing acetylated inulin microparticles. By using the spray drying technique, gallic acid (GA) was encapsulated in modified acetylated inulin starch [190]. The impact of acetylation of inulin on the encapsulation efficiency and release behavior of gallic acid in water was investigated. The introduction of acetyl groups into the starch molecules led to a greater GA encapsulating capacity while the same introduction of an acetyl group into inulin resulted in decreased GA encapsulation efficiency. Due to the structural difference between inulin and starch, the acetylation of starch results in structural alteration and stronger hydrogen bonding. In contrast, modification of inulin with acetyl group reduces the water solubility. The in vitro release profile demonstrates that inulin acetate was superior and better than starch acetate as a carrier for the delivery of gallic acid. The GA release profile was similar for both unmodified and acetyl starch. However, inulin acetate particles release GA at a lower rate compared to native unmodified inulin. A change in the physicochemical properties of the new derivative (decreased hydrophilic, solubility, and swelling ability) of modified inulin might be a reason for the change in release rate.

Another fascinating strategy used in the formation of microsphere from inulin derivatives is the solvent precipitation method. Acetylated and succinylated inulin were synthesized by reacting inulin with both acetic and succinic anhydride. Using solvent precipitation method, microsphere of inulin acetate and inulin acetate succinate with both drug and no drug were prepared [191]. Cationic compounds such as chlorhexidine and chymotrypsin were used as model drugs. The microspheres diameter varies from 0.5 to 4 μm for inulin acetate and inulin/chymotrypsin microspheres from 90 to 130 μm for inulin acetate/chlorhexidine microspheres. The in vitro drug release results show that the microspheres allow the extended release of the incorporated drugs. Another feasible approach in the formation of microsphere from inulin involves the coacervation technique. Water-soluble drug E, E-bis (aminobenzylidene) cycloheptanone [(E, E)-BABCH] was encapsulated using inulin and modified inulin acetate as starting material with and without 1,12-dodecanedicarboxylic acid [192]. The microspheres diameter was about 0.5 to 5 microns and the influence of variable parameters such as drug mass, speed, stirring, and centrifugation time on the encapsulation efficiency of the modified inulin was investigated. The in vitro release profile for unmodified inulin and inulin acetate shows burst release of about 58–62% within 5 min. However, the microsphere formed with 1,12-dodecanedicarboxylic acid reduce the burst release to about 32% within 15 min and allows controlled release of the remaining drug over 2.5 days. Inulin microparticles find application in colon targeting. Inulin microparticles were prepared from acetylated inulin for colonic delivery of indomethacin by electrospraying technique. The acetylation of inulin before microsphere formation improves the formation of inulin microspheres without trading off the colon targeting property of inulin [193]. The in vitro release of indomethacin under simulated GI conditions similar to the GIT shows that these microparticles were stable to acid degradation of the stomach environment, and about 60% of loaded indomethacin was only released in the simulated colonic fluid in the presence of inulinase after 18 h in the colonic fluid. The author concludes that indomethacin was release from the drug-loaded microparticles due to the degradation of the inulin glycosidic linkage by the inulinase microflora of the colon.

### 2.7. Inulin Nanoparticles

**Figure 9:**
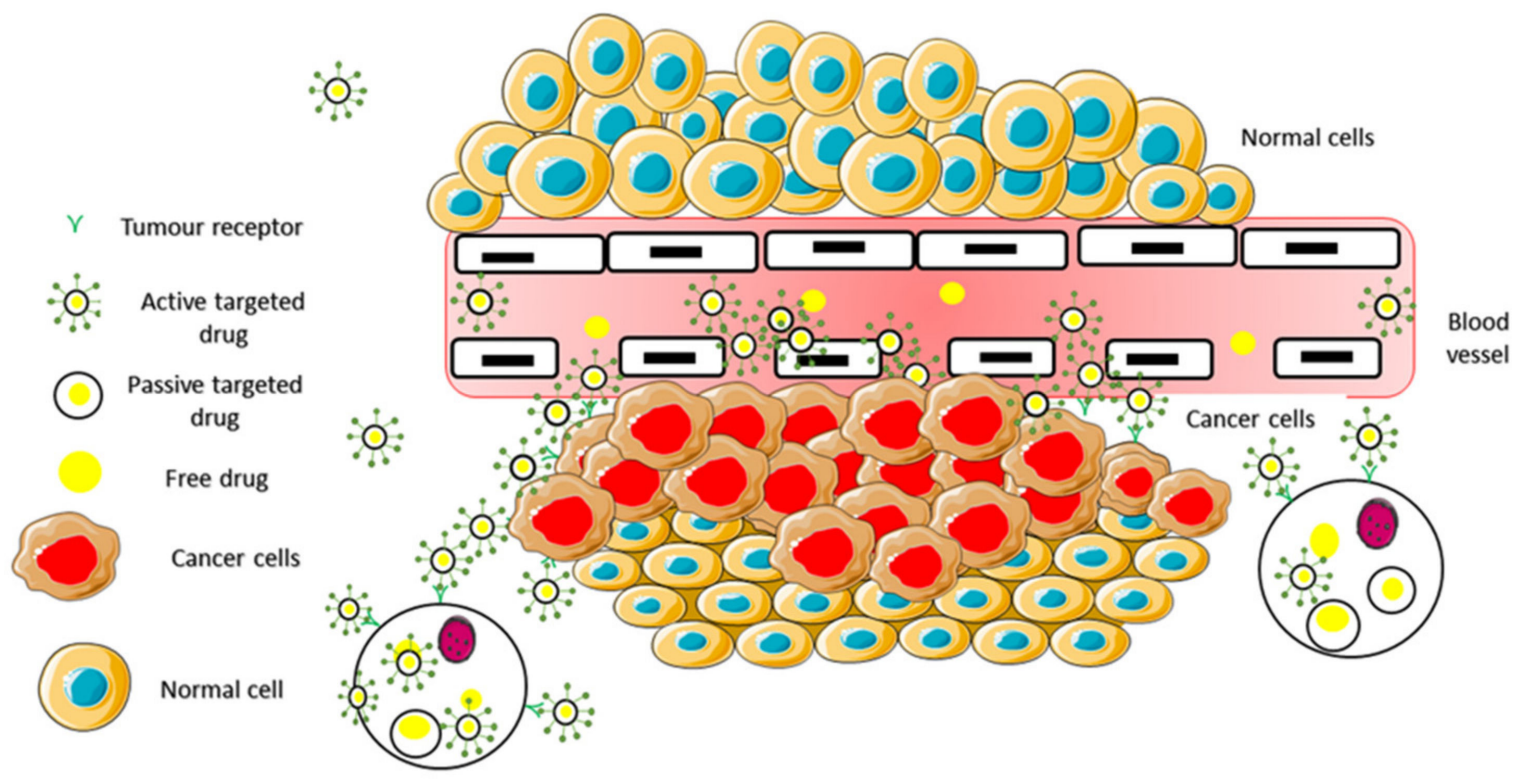
Active binding of the ligand attached to the nanocarrier surface to receptors commonly overexpressed by the cancer cells.

**Figure 10:**
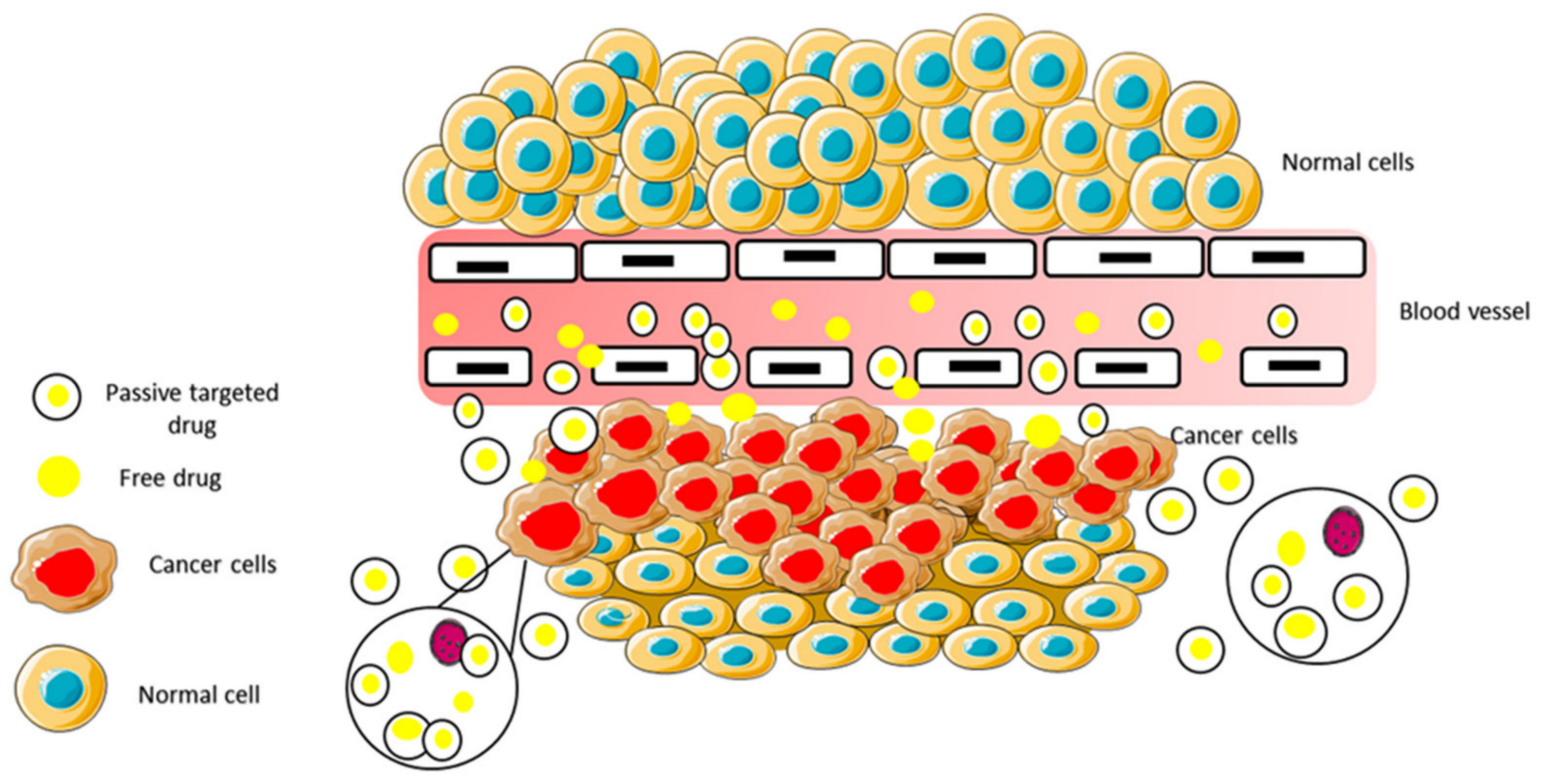
Passive targeting of cancer cells using nanoparticles via leaky endothelium.

Nanoparticles can target cancer cells passively via leaky endothelium (EPR) or actively by binding to specific receptors using ligands on the surface of the nanoparticles (Figure 9 and Figure 10) [194]. The size of nanoparticles controls the interaction of the nanoparticles with cells, trafficking as well as internalization. This translates to the delivery efficiency of the system.

#### 2.7.1. Active Targeting

##### Self-Assembling for Active Targeting

**Scheme 2:**
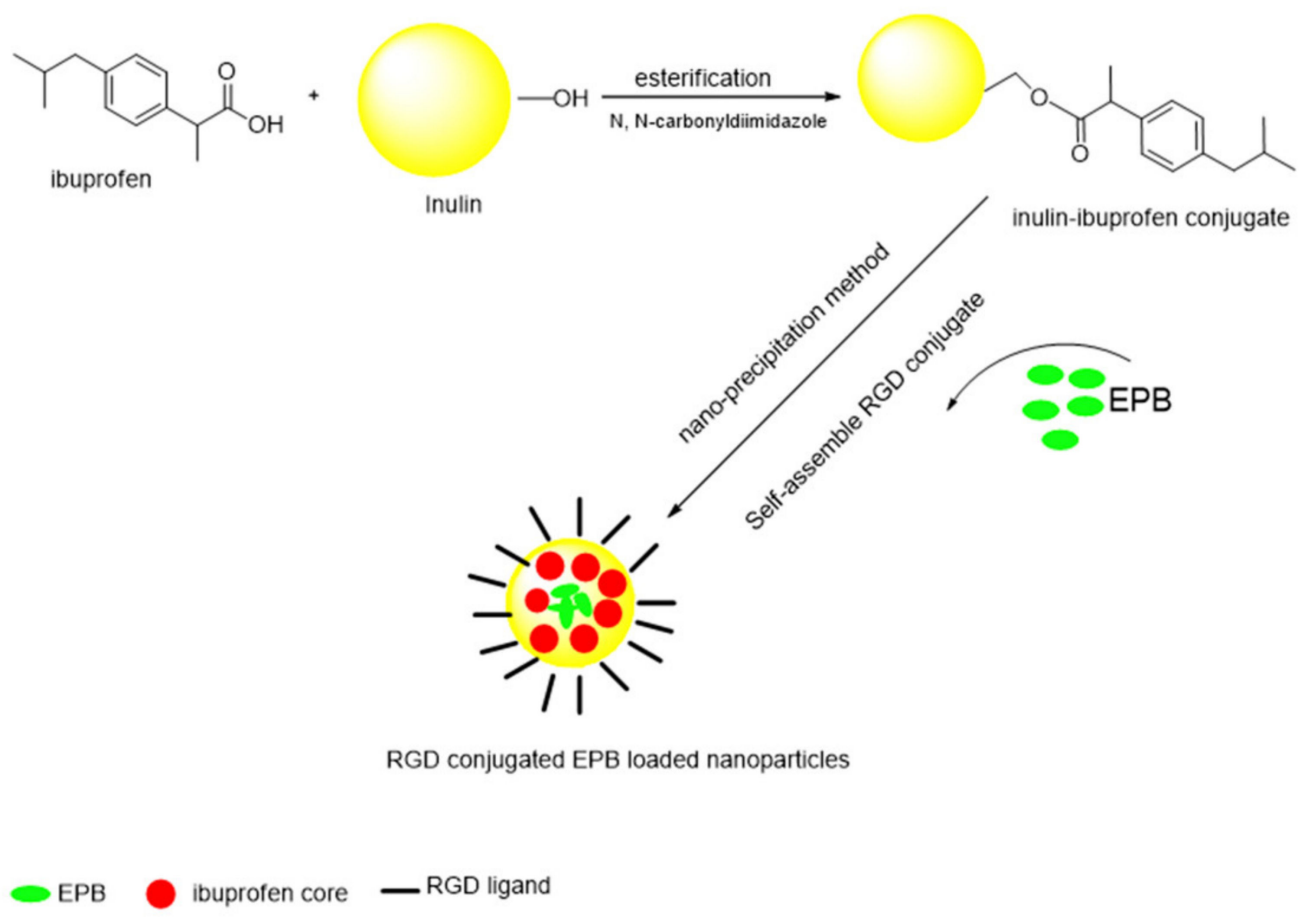
Formation of EPB loaded nanoparticles by self-assembly of inulin-ibuprofen [195].

The heterogeneous tumor’s extracellular matrix in cancer cells limits and prevents the accumulation of nanoparticles inside cancer cells. To overcome this challenge, Zhang et al. [195] developed RGD-peptide conjugated inulin-ibuprofen nanoparticles for the smart delivery of epirubicin. The capacity of the modified inulin–ibuprofen conjugate to undergo self-assembly into nanoparticles with inulin forming the hydrophilic shell and the ibuprofen forming the hydrophobic core was utilized to enhance the active targeting of the inulin-based nano-carrier in the delivery of epirubicin (Scheme 2). The chance of premature drug release outside the target site can be minimized by attaching targeting ligand such as RGD peptide to the surface of the nanoparticles. TEM analysis showed that both EPB loaded nanoparticles and RGD conjugated nanoparticles are round with a diameter of about 135 nm and 138 nm, respectively. The in vitro release studies revealed a pH-dependent pattern with slow-release at neutral pH 7.4 and significant release of EPB at the acidic environment (pH 5.0) of cancer and lysosomes. The conjugation of RGD-peptide to the EPB loaded nanoparticles improves the cellular uptake and cytotoxicity of the nanoparticles compared with EPB loaded nanoparticles. The result suggests that this approach may be useful in the smart delivery of drugs to solid tumors.

The use of folic acid as a ligand can also offer the specific targeting capability for the inulin derivative towards tumor cells. This strategy was exploited by Licciardi et al. [196]. Modified inulin derivative acted as a coating agent for gold nanoparticles loaded with DOX and functionalized with folic acid ligand to achieve targeted and selective delivery to MCF7 cancer cells. In this study, Inulin-2-aminoethyl-carbamate (INU-EDA) was conjugated with folic acid using 1-Ethyl-3-(3-dimethylaminopropyl) carbodiimide (EDC) and N-hydroxy succinimide (NHS) as coupling agents to form inu-FA derivative. This derivative was subsequently used to functionalize and coat the surface of 40 nm gold nanospheres. The interaction between the amino pendant group in the inulin-FA derivative and the gold helped to maintain and stabilize the nanoparticles. The characterization of the nanoparticles using DLS and UV showed that DOX was successfully adsorbed onto the surface of the gold nanoparticles due to electrostatic interaction and possibly the formation of the gold complex [AuCl(NH2R)] [197]. The authors reported a hydrodynamic diameter of 40 nm from the DLS result. This small size improve the targeting behavior leading to increased anticancer activity.

This approach of using folate ligand results in increased uptake of nanoparticles by MCF7 cell line overexpressing folate compared to folate receptor-deficient cell line such as human bronchial epithelial cells via receptor-mediated endocytosis. In addition to the cellular uptake, the targeting moiety folic acid ligand enhances the accumulation of the nanoparticles into the MCF7 cells.

#### 2.7.2. Passive Targeting

##### Gold Nanoparticles (GNPs)

To address the aggregation problem that often arises when citrate is used as a capping ligand [198]. Licciardi and colleagues [196] used inulin derivative (INU-EDA) and thiolated polyethyleneglycol (PEG-SH) as a coating agent and pre-stabilizing agent, respectively, for the fabrication of 40 nm gold nanospheres [199]. Characterization of the gold nanoparticles using TEM shows no change in particles size or evidence of aggregation after the modification. In addition, UV, DLS, and zeta potential analysis validate the stability of the DOX nanosystem in different media. The HPLC analysis results revealed that about 12% of DOX was loaded, and encapsulation efficiency was about 60%. The high encapsulation efficiency was attributed to physical interactions of inulin backbone INU-EDA and DOX and also to the hydrophobic interactions of DOX with the gold core. Using a co-culture cell model it was demonstrated that the nanosystem allowed the selective accumulation of the nanoparticles in malignant cancer cells. The authors suggested that the use of inulin derivative INU-EDA improved loading of DOX and stabilization of the Au@PEG-INU NPs in a buffer for 14 days. Interestingly, the nanosystem allowed slow, sustained, and selective release of DOX into cancer cells. Further potential application of this system in light-controlled drug release may be feasible because INU-EDA can be used for coating different sizes and shapes of gold nanoparticles.

##### Inulin Based Magnetic Nanoparticles

SUPERPARAMAGNETIC NANOPARTICLES (SPIONs)

**Figure 11:**
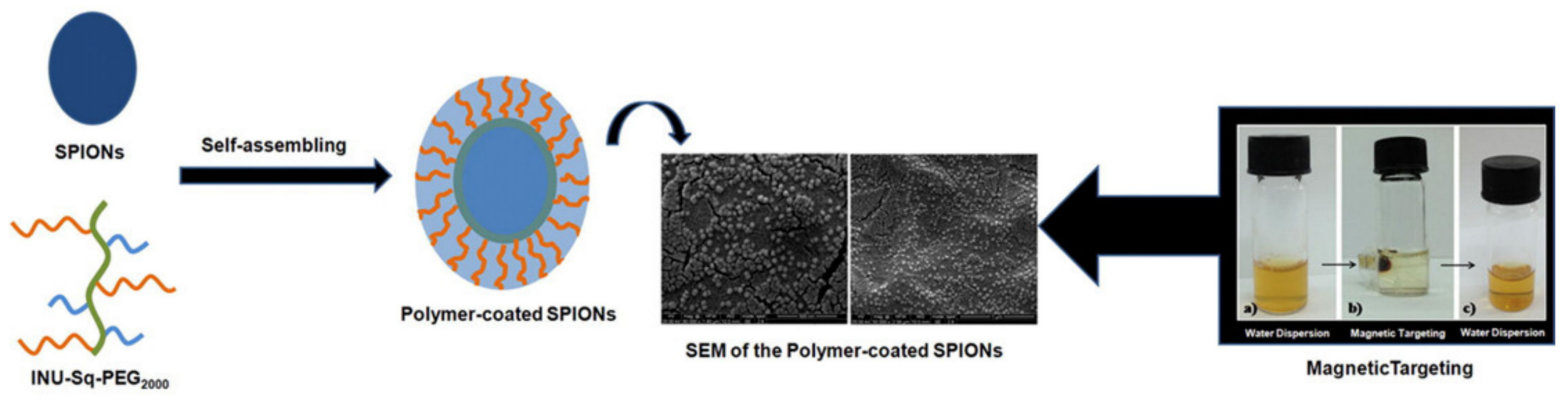
Inulin-based polymer coated SPIONs loaded with DOX. Reproduced with permission from [207] Copyright 2014, Elsevier.

One major challenge limiting the use of iron-oxide particles is the rapid elimination from the blood by the reticuloendothelial system (RES) [200,201]. Modification of the iron oxide particle is one of the effective strategies that can be employed to reduce RES clearance. The surface modification of iron oxide particles can improve stability, biocompatibility and allow multifunctional use of the particles as a platform for drug delivery [202] and imaging agent [203]. The magnetic nanoparticle can be used to address limitations such as invasive long-term treatment, treatment failure, tumor resistance, severe adverse effects, and non-selective accumulation to target cells [204,205]. SPION based drug delivery exploits a dual-targeting strategy (leaky structure of tumor vasculature-EPR and magnetic targeting to increase targeting efficiency [206]. SPION is superior to other drug delivery carriers as it allows imaging and drug delivery simultaneously. In addition, it offers better targeting efficiency. However, its use is limited by the formation of acidic by-products, which could result in inflammation and poor biodegradability. Suitable inulin derivatives have been used to overcome the drawbacks above. Inulin biocompatibility and biodegradability make inulin-coated superparamagnetic iron oxide nanoparticles a suitable platform for the delivery of drug and imaging agents. In addition, inulin improves stability and prolongs the blood circulation of SPION. Scialabba and co-workers synthesized amphiphilic inulin derivatives used for coating SPIONs [207]. This derivative, once placed in aqueous media, increases the loading of DOX significantly during the self-assembling process [207]. Characterization of the IC-SPIONs using SEM and DLS analysis confirmed the stability of the modified inulin with measurement of 21 ± 8 mV for zeta potential and spherical morphology a diameter of about 50 nm. The hydrophobic interaction between the aromatic ring of DOX and the squalenoyl appendage moieties leads to the formation of smaller nanoparticles, and this allows the loading of higher DOX (11.6 ± 0.5% (*w/w*)). In addition, the squalenoyl portion adds hydrophobicity to the inulin backbone, which assists in stabilizing the SPION coating via van der Waals forces. The release studies in two different media simulating the different human body compartments revealed a slow release pattern with about 12% of doxorubicin released after two days, irrespective of the pH of the media. The nanoparticle size (~55 nm) allowed the efficient uptake and internalization of the particles into the cytosol compartment area. Compared with free doxorubicin, the inulin-coated SPION showed three-fold anticancer activity under the control of an external magnetic field. The inulin coated SPION allow preferential accumulation and release of DOX with the external magnetic field (Figure 11).

In a similar study, the versatility of inulin derivatives was exploited to obtain carboxymethyl inulin (CMI). It was subsequently used for coating iron oxide for use as a magnetic fluid hyperthermia agent [208]. This work involves coating the surface of iron oxide particles decorated with amine silane with carboxylmethyl inulin using amide covalent linkage. Physicochemical characterization using different techniques such as FTIR, SEM, DLS, and SQUID magnetometry analysis showed evidence of new band appearance, hydrodynamic diameter 70 ± 10 nm, and polydisperse particles. The inulin coated iron nanoparticles were not only less toxic to five different cancer cells despite their higher concentration of 1.5 mg/mL, but also a significant amount was uptaken by Caco-2 cells. Moreover, the amide attachment of CMI through the APS layer of the iron oxide gives better stability to the nanosystem when stored in water and other media. The fact that no change in hydrodynamic diameter with change in temperature (25 °C and 60 °C) was observed makes this nanosystem a potential magnetic fluid hyperthermia agent.

##### Functionalized Superparamagnetic Nanoparticles

To increase the active targeting capacity of inulin nanoparticles towards specific cancers cell that over-express folic acid on the surface, Scialabba et al. developed an amphiphilic inulin derivative using folic acid as a ligand, which was subsequently used as a coating material for SPIONs [209]. Folic acid is over-expressed in various human cancers such as colon, breast, lung, and ovary and prostate cancer. This encourages the engineering of an efficient drug delivery platform capable of selectively binding to the target cell. In this work, a new amphiphilic graft copolymer INU-LA-PEG-FA copolymer, which can self-assemble in aqueous media, was synthesized by a reaction that involves two steps. First, lipoic acid (LA) was attached to the inulin backbone, followed by conjugation with folic acid via PEG spacer by reacting with NH2-PEG-FA. The final copolymer INU-LA-PEG-FA copolymer was then employed as a coating and stabilizing agent for SPION, and the surface is enriched with folic acid to improve the selective targeting of cancer cells. The change in the size from 14 to 131 nm as well as a surface charge from ~−30 to −5 Mv promote and helped the successful adsorption of the copolymer to the surface of the SPION. The cell viability and erythrolysis studies showed that FA-SPIONs were not toxic to different cancers cells (HCT116, MCF7). In addition, the in vitro and in vivo MRI experiments revealed that FA-SPIONs were efficient as a contrasting agent for MRI analysis combined with superior targeting ability attributed to the surface ligand folic acid. The use of PEG spacer prolonged the half-life of the drug delivery system by reducing their removal by the reticuloendothelial system.

##### Magnetoplexes

The development of new and tunable RNA and DNA delivery platforms for the treatment of gene-related disorders is drawing attention after the approval of Glybera by the European Medicines Agency (EMA) [210]. Nevertheless, low transfection efficiency, unloading of the siRNAs to the target site, poor siRNAs uptake, and degradation limit the potential use of RNA and DNA by most routes [211,212]. For this reason, the use of non-viral vectors for the delivery of RNA and DNA is becoming popular due to the low cost of production, safety, less immunotoxicity, and low pathogenicity when compared to the viral approaches. Apart from the drug and ligand conjugation, the hydroxyl group of inulin serves as an anchor in the formation of polycations derivative that can easily complex with the negatively charged siRNA and DNA. Inulin derivatives have been used in coating magnetic nanoparticles in the delivery of therapeutic genes. In one approach, inulin derivative was used for dual purposes (coating SPION and complexation with siRNA). Licciardi et al. enhanced siRNA delivery by developing SPION stabilized with inulin-ethylenediamine derivative obtained by enhanced microwave synthesis (EMS) [213]. The inulin coated SPIONs were less toxic to 16HBE and HCT116 cancer cell lines, and at the same time, their transfection efficiency was significantly increased. The cellular uptake and accumulation of the siRNA in the cells were also enhanced in the presence of an external magnet [213].

##### Self-Assembled Inulin Nanoparticles

Zhang and co-workers developed an amphiphilic inulin derivative by the esterification reaction between the functional hydroxyl group of inulin and the carboxylic acid groups of ibuprofen using carbodiimide chemistry. The inulin was subsequently self-assembled into nanoparticles by the nanoprecipitation method [214]. The amphiphilic inulin derivative self-assemble into nanoparticles with a hydrophobic core and hydrophilic shells in an aqueous environment. By chemically attaching hydrophobic moiety such as ibuprofen to inulin hydrophilic backbone allows the formation of self-assembled nanoparticles (SNP). The authors used a pyrene fluorescence probe to confirm the formation of nanoparticles by investigating critical aggregation behavior. The physicochemical characterization of the nanoparticles was conducted using ^1^H NMR, TEM, and DLS, and it was confirmed that ibuprofen was attached to the inulin backbone, with a hydrodynamic diameter of 141 nm for the drug-loaded nanoparticles with spherical shape nanoparticles. In a similar study, methylprednisolone was enveloped into the inner hydrophobic core of the nanoparticles, and the in vitro release in phosphate buffer solution by dialysis method showed a sustained release pattern with about 94.9% of the drug released in 4 days. The cell viability studies showed that both empty nanoparticles and drug-loaded methylprednisolone nanoparticles were less toxic to the Rat Schwann cell line RSC-96. It should be emphasized that this drug-loaded nanoparticle may be utilized in the smart and targeted delivery of drugs such as methylprednisolone for spinal cord injury.

##### Nanostructured Carbon-Nanosheets

**Figure 12:**
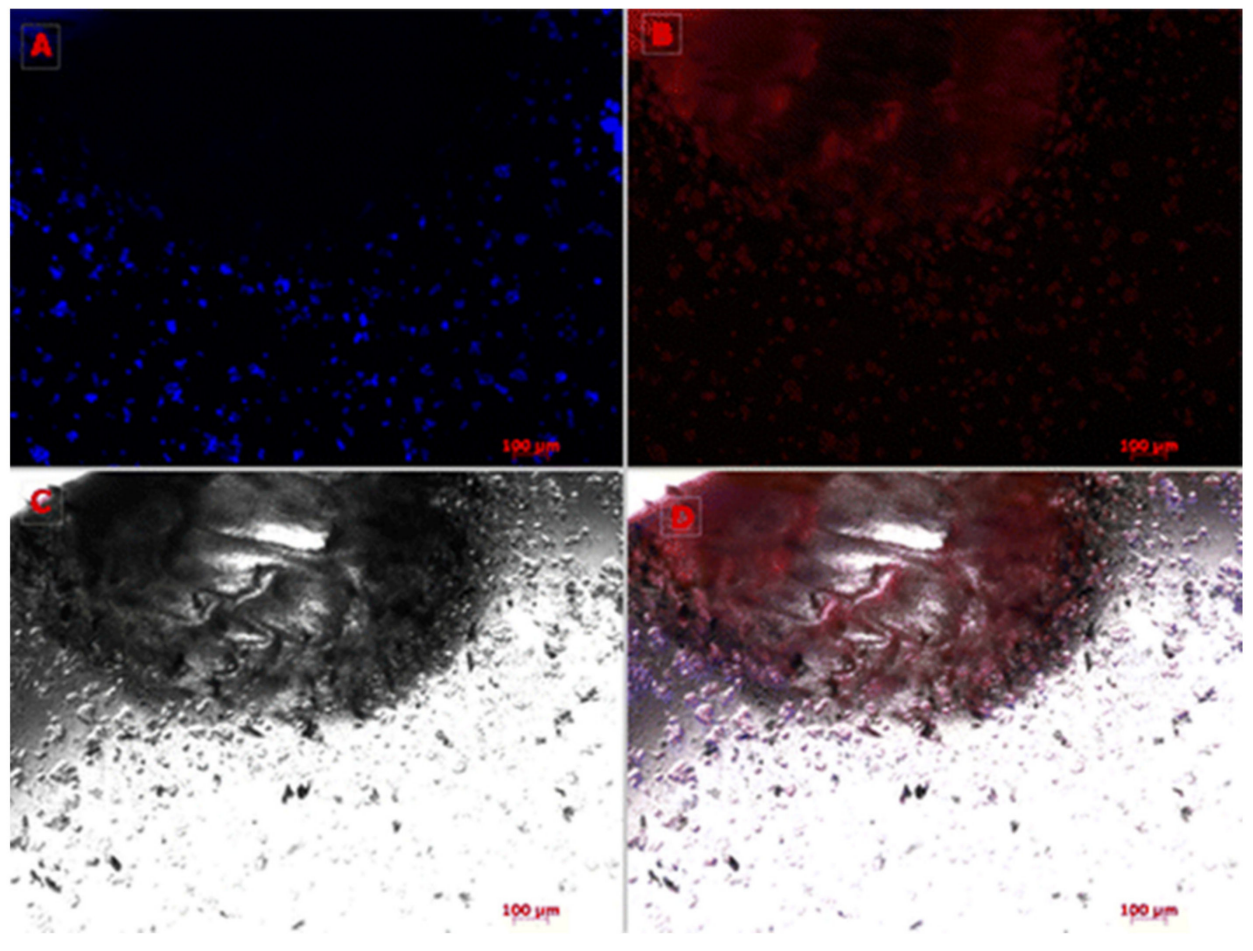
Thermoablation of RGO/CJ-PEGBT treated MCF-7 cells obtained with an 810 nm laser fitted at 7 × 10^–2^ W mm^–3^: blu nuclei (**A**), red doxorubicin (**B**), bright field (**C**), merging of all fluorescence (**D**); magnification 5×. Reproduced with permission from [215]. Copyright 2015, American Chemical Society.

The quest for a multifaceted approach to the improvement and treatment of cancer was the compelling reason and motivation for the development of a nanosystem with RGO-induced hyperthermia and a smart chemotherapy delivery system (Figure 12). Inulin-doxorubicin prodrug conjugate (INU-EDA-P, C-Doxo) with proven pH sensitivity and self-assembling properties was functionalized with PEG-biotin using Huisgen cycloaddition click chemistry [215]. The use of Huisgen cycloaddition click chemistry to functionalize inulin prodrug (INU- EDA-P, C) is a classic example of using molecular designs and modification reactions to form novel polymeric materials with advanced function.

In this case, the functionalizing of the inulin derivative with biotin allowed preferential selectivity of the delivery system towards cancer cells. The available alkyne groups in the inulin prodrug (INU- EDA-P, C) support conjugation of the prodrug with biotin, which is the targeting agent via the PEG spacer arm. The addition of the reduced graphene oxide (RGO) and biotinylated Inulin-doxorubicin conjugate results in a Π-Π-staked nanosystem with a synergic anticancer effect. The stability, self-assembling formation of the stable π-staked system was confirmed using UV, AFM, and DSC analysis. It was easy to control the amount of DOX loaded, which is an important factor that can influence the efficacy of nanoparticles in vivo by varying the ratio of conjugate/RGO. The biotinylated system shows both higher efficacy and potency compared with the non-targeted system, which is attributed to preferential and enhanced uptake as well as efficient drug release at acidic tumor pH. The application of an 810 nm laser diode cold laser beam resulted not only in the release of DOX but also physically burnt/damaged the cells. This was evidenced by a decrease in cell viability and blocked cell proliferation. The result demonstrated the dual ability of this nanosystem to function as a thermoablation agent with a hyperthermia-triggered drug release mechanism. This nanosystem could significantly broaden the use of inulin nanoparticles in both drug delivery and biomedical fields.

### 2.8. Solid Dispersion

**Figure 13:**
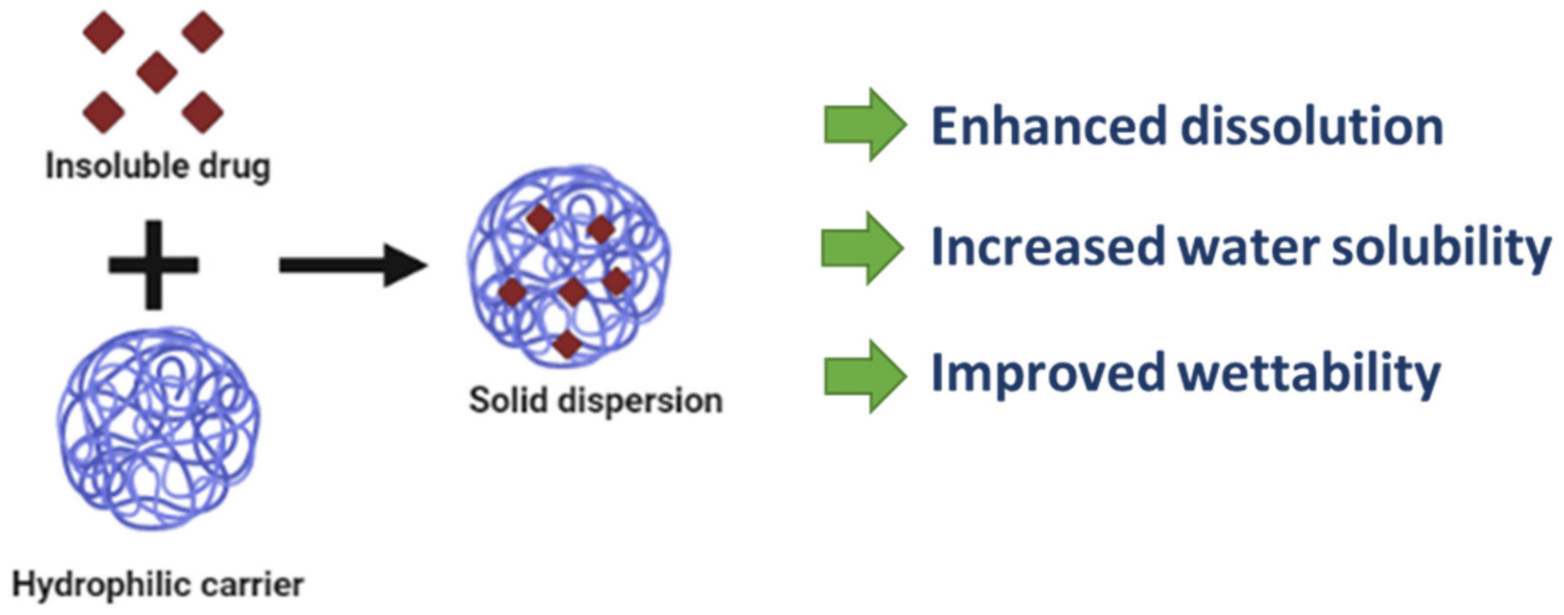
The formation of solid dispersion and the benefits.

Increasing both the dissolution rate and bioavailability of drugs with poor water solubility (Biopharmaceutics Classification System class II and IV) remains a challenging task for formulation scientists. One way of dealing with this problem involves the use of solid dispersions (SDs) (Figure 13). Drugs with low and poor water solubility may be dispersed in solid dispersion by using a hydrophilic carrier, which helps to increase the surface area of the drug available for dissolution, improved drug wetting, reduce particles sizes of drugs and improve the solubility of drugs under physiologically relevant conditions [216].

Hydrophilic carriers such as inulin can be utilized as solid dispersion carriers to improve and enhance the solubility of drugs. The use of inulin solid dispersion technology to boost the release and bioavailability of poorly soluble drugs such as nifedipine (218), diazepam [217,218,219], lipophilic drugs [218], (fenofibrate, ritonavir, and efavirenz) cyclosporine A [220], curcumin, irbesartan [221], and itraconazole have been reported. The high glass transition temperature of inulin makes it a suitable carrier in solid dispersion formulation because this allows stability of the final formulation. Inulin glassy SD improved the stability of labile THC D9-tetrahydrocannabinol [222], diazepam [218,223], and fenofibrate [218] due to the high glass transition temperature of inulin. The dissolution of the carrier results in faster hydration of the poorly water-soluble drugs. One of the limitations with a sugar glass solid dispersion system is low drug release due to crystallization after exceeding certain drug load percentages with the use of sugars such as sucrose or trehalose [219]. However, compared to other simple sugars such as sucrose or trehalose as a carrier for solid dispersion, inulin was a better candidate because it exhibits slower dissolution and a higher crystallization threshold [219]. The superiority of inulin over polyvinylpyrrolidone (PVP) as a carrier for solid dispersion for different drugs with poor water solubilities such as diazepam, ritonavir, and efavirenz was demonstrated [218]. Inulin lauryl carbamate (Inutec SPI) is a surfactant formed by covalently attaching lipophilic alkyl side chains to the hydrophilic inulin backbone. The use of Inutec SPI as SD permits the loading of a high amount of drug without crystallization during dissolution. The inulin derivative showed superior dissolution behavior and improved stability of the final formulation due to its surface-active nature. The use of Inutec SPI solid dispersion outperformed pure itraconazole because solid dispersion carriers of itraconazole yield better result outcomes [224]. The dissolution behavior, releases, and bioavailability for a class IV drug TMC240 (HIV protease inhibitor characterized by poor solubility and permeating properties) were significantly improved by using Inutec SP1 solid dispersion technology [225]. Apart from Inutec SPI, the use of derivatives with different moieties attached to the inulin backbone as solid dispersion for dissolution enhancement is possible. The dissolution and bioavailability of irbesartan were increased by using poly (acrylic acid) grafted inulin copolymer [221]. The inulin backbone was grafted with hydrophilic moieties, which leads to a higher dissolution rate and bioavailability of water-insoluble irbesartan. The incorporation of poorly soluble HTC into the inulin solid dispersion matrix not only increases the stability but also the bioavailability [226]. The stable dry powder obtained with glassy inulin polymer allowed the development of a more convenient sublingual dosage formulation with immediate release within 3 min compared to about half an hour for a tablet.

There is a high probability of undesired recrystallization with higher drug loading with solid dispersion technology, which can be minimized with the addition of surfactant [37]. Interestingly, despite the high drug loading, this problem did not arise with solid dispersions of diazepam [219] and cyclosporine A [220]. Inulin solid dispersion of cyclosporine A (CSA) prepared by spray freeze-drying surpass formulation with a physical mix of both pure CSA and spray freeze-dried inulin even at higher drug loading [220]. The solid dispersion results in the formation of large porous particles deemed suitable for the pulmonary route due to deep lung deposition and high emitted dose. The use of inulin in the SD increased the specific surface area and wettability of CSA, making it a suitable carrier for the design of pulmonary formulation useful for the treatment of lung transplant patients. The superior dissolution enhancement properties of inulin as solid dispersion can be ascribed to different mechanisms such as the formation of flower-like platelets of inulin around the drug [221,227], surface-active nature of the inulin derivative [218] as well as higher surface area and improves wetting of drugs agents [220]. Another important factor that can influence drug dissolution and release of solid dispersion is the chain length of inulin used in the formation of the solid dispersion. Drug dissolution from solid dispersion may be hindered by using inulin with higher molecular weight. The higher molecular weight inulin does not give better solid dispersion because of the slower dissolution of the inulin matrix. Lower molecular weight inulin due to the oligomeric nature performed better than higher molecular weight, as demonstrated with solid dispersion of tacrolimus with 1.8 kDa and 4 kDa inulin [228]. The smaller chain 1.8 kDa has higher solubility in an aqueous environment compared to the longer 4 kDa inulin chain [219]. It is possible to use solid inulin dispersion as a suitable carrier for incorporating higher rovement in the dissolution and physical stability of formulations.

The comprehensive summary of the different inulin drug delivery systems is provided below in Table 1.

## 3. Toxicological/Clinical Studies Performed Related to Inulin or Inulin-Containing Products

There is limited data on clinical trials of drug formulations containing inulin. However, several reports on the use of inulin microparticles, specifically Advax as vaccine adjuvant in different vaccines, indicate that delta inulin was well tolerated and safe [178,183]. There is no toxic reaction or concern with the safety profile. The use of these inulin microparticles in vaccines tested in humans was found to be safe. This excellent safety profile makes it a good and potential candidate for vaccine adjuvant. Recently, inulin microparticles were utilized as an adjuvant for SARS-CoV-2 vaccine (COVID Vaccine), which is currently moving into human clinical trials.

## 4. Conclusions and Expert Opinion

This review highlighted the application of inulin as a drug carrier. Inulin possesses good biocompatibility, biodegradability, molecular flexibility, membrane-stabilizing effect, immune modulation effect, and easy chemical modification due to the hydroxyl group. This makes inulin an excellent candidate as a carrier for drug delivery systems and biomaterials. The scope of its use can be increased via modification of its chemical structure. The hydroxyl group serves as an anchor and active site for the modification of inulin. This further allows the fabrications of different inulin drug delivery systems such as hydrogel, micelles, prodrugs, liposomes, solid dispersion, microparticles, and nanoparticles. The exceptional tropism of inulin towards the kidney allows targeted delivery of drugs to the kidney. The colon targeted delivery is possible since inulin is only hydrolyzed by inulinases produced by Bifidobacterium in the colon. The hydroxyl group also confers in inulin good swelling in water when utilized for fabrication of hydrogels. The microparticles and nanoparticle size range permit efficient uptake by the macrophages and monocytes. By functionalization of the existing inulin derivatives with targeting ligands such as biotin, folic acid, and RGD-peptide, the targeting efficiency can be improved. The use of solid dispersion constructed with inulin allows the improvement of the solubility and bioavailability of drugs with poor water solubility. It is worth mentioning that Inulin’s potential application for light-controlled, lysosome-triggered, and pH-targeted delivery needs further investigation.

## Figures and Tables

**Table 1:** Showing a summary of the different delivery systems, drugs delivered, method of fabrication and key outcomes.

Delivery System	Drug Delivered	Method	Key Outcomes
Hydrogels and Beads, Vesicles, Pellets, tablets, and nano-hydrogel	NSAIDs, hormones, peptides, corticosteroids, anticancer, immunoglobulin G (IgG), bovine serum albumin, and lysozyme, MTX, 5-aminosalicylic acid, probiotics strains	radical polymerization radical copolymerization Michael addition crosslinking UV radiation, chemical crosslinkers solvent precipitation	Allow targeted and localized delivery of drugs to the colon
Micelles	curcumin and celecoxib rifampicin doxorubicin	Self-assembly micelles using modified inulin Amphiphilic micelles	Delivers high payload to cancer cells Use of biotin can also improve targeting
liposomes	Quercetin	Shealth Coating	Stabilizes liposome Coat liposome Liposome uptake marker
prodrugs	Metronidazole Procainamide 5-ASA	Conjugation chemistry ester and Schiff base prodrug	Improve stability Increase plasma half-life
Complexes/chelating agent	Gd–inulin complex iron supplements	Chelation and complex formation	Better contrasting agents
Microparticles	Vaccine adjuvants Isoniazid, pyrazinoic acid Doxorubicin Gallic acid Chlorhexidine Indomethacin	Conjugation chemistry coacervation technique Spray drying	Better cellular uptake Targeted delivery to infected cells
Nanoparticles Gold nanoparticles Supermagnetic nanoparticles (SPION) Self-assembly Nanostructured carbon sheet	Ibuprofen Doxorubicin Methylprednisolone	Modification of inulin via chemistry	Attaching ligand such as RGD, biotin improves cellular uptake Better targeting to cancer cells
Solid dispersion	Nifedipine Diazepam, fenofibrates Ritonavir, itraconazole, curcumin, Irbesartan	Inulin as hydrophilic polymer	Result in high loading without recrystallization Improve dissolution and physical stability

## Data Availability

Not applicable.
